# Comparative long-term outcomes of first-line CDK4/6 inhibitors plus endocrine therapy versus endocrine therapy in patients with HR+/HER2-metastatic or advanced breast cancer: a meta-analysis

**DOI:** 10.3389/fphar.2025.1600892

**Published:** 2025-07-25

**Authors:** Xiaojian Wang, Xinyi Liang, Chenchen Li, Mengtian Qin, Jianyu Wu, Yuechen Qin, Yue Zou, Haijian Zeng, Chunlan Li, Xiaomeng Huang, Haiyun Tao, Jieru Quan, Xiao Wang

**Affiliations:** ^1^ The First Affiliated Hospital of Guangxi University of Science and Technology, Guangxi University of Science and Technology, Liuzhou, Guangxi, China; ^2^ Department of Medical Oncology, The Sixth Affiliated Hospital, Sun Yat-sen University, Guangzhou, Guangdong, China; ^3^ School of Economics and Management, Guangxi University of Science and Technology, Liuzhou, Guangxi, China

**Keywords:** breast cancer, metastatic, endocrine therapy, CDK4/6 inhibitors, progression-free survival, overall survival, objective response rate, meta-analysis

## Abstract

**Introduction:**

This meta-analysis was designed to compare the long-term outcomes of first-line cyclin-dependent kinase 4/6 (CDK4/6) inhibitors plus endocrine therapy (ET) versus ET in patients with HR+/HER2-metastatic or advanced breast cancer (BC).

**Materials and methods:**

Four databases (Medline, Embase, Web of Science, and CENTRAL) were searched for literature comparing First-line CDK4/6 inhibitors plus ET to ET in patients with HR+/HER2-metastatic or advanced breast cancer. The search was conducted from the database’s establishment to April 3, 2025. Progression-free survival (PFS), overall survival (OS), objective response rate (ORR), disease control rate (DCR), and severe treatment-related adverse events (SAEs) were subjected to meta-analyses.

**Results:**

Twelve Randomized controlled trials (RCTs) were included in the meta-analysis. The meta-analysis included a group of 4,957 patients diagnosed with HR+/HER2-metastatic or advanced breast cancer. Within this cohort, 2,877 patients were administered First-line CDK4/6 inhibitors together with ET, while 2080 patients received first-line ET alone. Compared with ET, First-line CDK4/6 inhibitors plus ET yielded superior ORR (RR = 1.39, 95% CI, 1.28 to 1.51, P < 0.01), DCR (RR = 1.09, 95% CI, 1.05 to 1.14, P < 0.01), PFS (HR: 0.57, 95%CI 0.53 to 0.62, P < 0.01) and OS (HR: 0.81, 95%CI 0.73 to 0.89, P < 0.01). Reconstruction of Kaplan-Meier curves for OS and PFS using the IPDformKM program provided a clear and comprehensible representation of oncological outcomes. First-line CDK4/6 inhibitors plus ET was more effective than ET in terms of PFS (median survival time: 27.0 months versus 14.4 months, HR: 0.55, 95%CI 0.51 to 0.59, P < 0.01) and OS (median survival time: 59.6 months versus 50.0 months, HR: 0.79, 95%CI 0.72 to 0.87, P < 0.01). With regards to safety, First-line CDK4/6 inhibitors plus ET exhibited a greater likelihood of encountering SAEs (RR = 1.54, 95% CI: 1.30 to 1.82, P < 0.01) in comparison to ET.

**Conclusion:**

The present meta-analysis reported comparative long-term outcomes of CDK4/6 inhibitors plus ET versus ET as first-line therapy for HR+/HER2-metastatic or advanced breast cancer. Compared with ET alone, CDK4/6 inhibitors plus ET as first-line therapy provided improved ORR, DCR, PFS, and OS. Furthermore, the heightened efficacy of CDK4/6 inhibitors plus ET was accompanied by a rise in SAEs.

**Systematic Review Registration:**

https://www.crd.york.ac.uk/PROSPERO/view/CRD42024590572, Identifier CRD42024590572.

## 1 Introduction

Breast cancer (BC) is the most often detected malignant disease in women and a significant cause of cancer-related deaths globally. It is projected that there will be 2.3 million new cases of BC in 2022, representing 11.6% of all documented cancer cases (Bray et al., 2024). The projected future prevalence of breast cancer (BC) is expected to surpass 3 million new cases and result in 1 million fatalities by the year 2040 (Cancer Gen ome Atlas Network, 2012; Arnold et al., 2022). The presence of three tumor markers—estrogen receptor (ER), progesterone receptor (PR), and HER2 status—primarily determines the classifications of breast cancer. Hormone receptor (HR) status is the sum of ER and PR evaluations. There are four main molecular subtypes that can be approximated by the joint HR/HER2 status: HR+/HER2− (roughly corresponding to the Luminal A subtype), HR+/HER2+ (Luminal B), HR−/HER2+ (HER2-enriched), and HR−/HER2− (triple-negative) (Clarke et al., 2012; Howlader et al., 2014; Kohler et al., 2015). Breast cancers that are positive for hormone receptors and negative for HER2 are the predominant subtype within every racial and ethnic group (Giaquinto et al., 2022).

Statistical data indicates that approximately 30%–40% of those who are diagnosed with early-stage breast cancer (BC) eventually progress to advanced breast cancer (BC) (Cardoso et al., 2018a), with a small proportion of patients already having distant metastases (Cardoso et al., 2012). The standard recommendation for first-line treatment of HR+/HER2-breast cancer (BC) patients is endocrine therapy (ET), which includes aromatase inhibitors (AI) or fulvestrant, unless there is a visceral crisis or life-threatening risk (Cardoso et al., 2018b; Rugo et al., 2016). Nevertheless, most individuals develop acquired resistance during endocrine therapy (ET), whereas a subset of patients may not react to the first medication (*de novo* resistance) (Milani et al., 2014) or have illness progression throughout treatment also because of acquired resistance (Sestak et al., 2013). Insufficient efficacy of single-agent endocrine therapy (ET) and the emergence of primary or secondary medication resistance make endocrine monotherapy increasingly inadequate for first-line treatment.

Novel cyclin-dependent kinase 4/6 inhibitors (CDK4/6) have revolutionized the treatment of many malignancies (Mouabbi et al., 2021). Dysregulated cellular division, a hallmark of cancer, is a pathogenic mechanism that promotes aberrant cell proliferation associated with malignancy. To progress from the G1 to the S phase of the cell cycle, it is essential to either hyperactivate cyclin-dependent kinase (CDK) 4 and 6 or deactivate the retinoblastoma protein (RB1) pathway (Gelbert et al., 2014). Over the past decade, the most significant advancement in the management of HR-positive, HER2-negative advanced or metastatic breast cancer (BC) has been the incorporation of CDK4/6 inhibitors in combination with endocrine therapy (ET) in international treatment guidelines, both as initial therapy and after disease progression following ET (Finn et al., 2015; Pernas et al., 2018; Spring et al., 2019). The US Food and Drug Administration (FDA) and the European Medicines Agency (EMA) have granted licenses for the use of three highly selective CDK4/6 inhibitors—palbociclib (PD0332991), ribociclib (LEE011), and abemaciclib (LY2835219)—to treat HR-positive advanced or metastatic breast cancer (Piezzo et al., 2020). Dilipiclib (SHR6390) is a recently developed orally delivered, selective inhibitor of CDK4/6 (Long et al., 2019; Wang et al., 2017). There is currently a growing interest among academics and practitioners in investigating the effectiveness and safety of combining various CDK4/6 inhibitors with hormone treatments (Turner et al., 2017).

The objective of this study was to provide evidence-based recommendations for clinical decision-making by conducting a meta-analysis to compare the long-term outcomes of First-line CDK4/6 inhibitors plus endocrine therapy versus endocrine therapy in patients with HR+/HER2-metastatic or advanced breast cancer.

## 2 Materials and methods

### 2.1 Search strategy

In compliance with the 2020 guidelines of the Preferred Reporting Project for Systematic Review and Meta-Analysis (PRISMA), the current meta-analysis was conducted. The present work has been officially recorded at PROSPERO under the registration number CRD42024590572. A systematic search was conducted in four databases, namely, PubMed, Embase, Web of Science, and the Cochrane Library, to identify literature published up to April 3, 2025. The search strategy used a combination of MeSH and free-text words following the PICOS principle. The search keyword was “breast cancer” AND “metastatic” AND “First-line” AND “CDK4/6 inhibitors” AND “endocrine therapy” AND “Randomized controlled trial”.

Supplementary Material-1 provided a comprehensive listing of the search results.

### 2.2 Inclusion and exclusion criteria

The criteria for inclusion were as follows: (1) patients with untreated metastatic or advanced HR+/HER2-breast cancer; (2) Patients in the intervention group received CDK4/6 inhibitors plus endocrine therapy; (3) patients in the controlled group received any endocrine therapy as their first-line medical treatment; (4) at least one of the following outcomes was reported: ORR, DCR, PFS, OS, and SAEs. (5) Study types: randomised controlled trials (RCTs). CDK4/6 inhibitors included Abemaciclib, Palbociclib, Ribociclib, and Dalpiciclib. Although Dalpiciclib has not been approved by the FDA and EMA, it was also included in this meta-analysis.

The criteria for exclusion were as follows: (1) different types of papers, such as case reports, publications, letters, comments, reviews, meta-analyses, editorials, animal studies, protocols, conference, etc; (2) other types of malignancies or diseases; (3) not relevant; (4) full text not available; (5) duplicate patient cohort.

### 2.3 Selection of studies

Selection of studies, including elimination of duplicates, was undertaken using EndNote (Version 20; Clarivate Analytics). An initial search was undertaken by two reviewers who independently deleted duplicate entries, assessed the titles and abstracts for relevantce, and classified each study as either included or excluded. The settlement was arrived at through the attainment of consensus. A third reviewer of the review would take on the role of an arbitrator if lacking a consensus.

### 2.4 Data extraction

Two independent reviewers performed a meticulous analysis of the title and abstract, followed by a detailed perusal of the whole text. In the case of a lack of data in the research report, results were requested by sending an email to the given contact person. The request date was recorded in EndNote. To address the inconsistencies, a third reviewer was consulted. The data collected includes the name of the first author, publication year, study area, trial ID, study design, sample size, intervention, participant age, trial phase, study period, median follow-up duration, ORR, DCR, PFS, OS and SAEs.

### 2.5 Risk of bias assessment

Using the Cochrane Collaboration risk of bias assessment tool, two reviewers independently evaluated the methodological quality of each individual study (Higgins et al., 2011). This study assessed six key aspects: randomization, allocation concealment, blinding procedures, attrition rate, result reporting, and other sources of bias. The quality assessment results determined the labeling of each feature as low, unclear, or high risk. The quality bias assessment was conducted by two writers, and any discrepancies were resolved through consultation with a third senior reviewer. RevMan version 5.3 (Review Manager, The Cochrane Collaboration, 2020) was used to graphically depict the summary of bias risk.

### 2.6 Evidence certainty

The certainty of evidence for the systematic review was assessed by two independent reviewers using the GRADEpro GDT (Prasad, 2024): GRADEpro Guideline Development Tool [Software]. In case of any discrepancy, a consensus was formed by mutual discussion with other reviewers.

### 2.7 Statistical analysis

The selection duplicate removal of studies included was conducted using EndNote (Version 20; Clarivate Analytics). The statistical analysis was performed using the Stata/SE version 12.0. The fixed-effects model incorporates the Relative Risk (RR) for Binary categorical outcomes in its initial structure. These metrics were reported together with their corresponding 95% confidence intervals (CI). The choice between fixed - effect and random - effect models was based on the I^2^ value and chi-square test P value. When heterogeneity was high (I^2^ >50%), the random - effect model was used. When heterogeneity was low (I^2^ ≤ 50%), the fixed - effect model was applicable. Statistical significance is defined as a p-value less than 0.05. Stata/SE version 12.0 was used to graphically depict the a overall hazard ratios (HR) for PFS and OS, we used funnel plot, Begg’s and Egger’s method to evaluate the publication bias regarding all indicator results (ORR, DCR, PFS, OS and SAEs).

In addition, Extraction of survival information was performed on PFS and OS Kaplan-Meier curves using the GetData Graph Digitizer. Data were subsequently rebuilt on an individual basis using the IPDformKM software. Reconstruction of the individual patient data and subsequent fitting of the survival data were performed using Guyot’s approach (Liu et al., 2021; Guyot et al., 2012). Next, the GraphPad Prism and MedCalc programs were used to create Kaplan-Meier graphs for PFS and OS statistics.

## 3 Results

### 3.1 Search results

**FIGURE 1:**
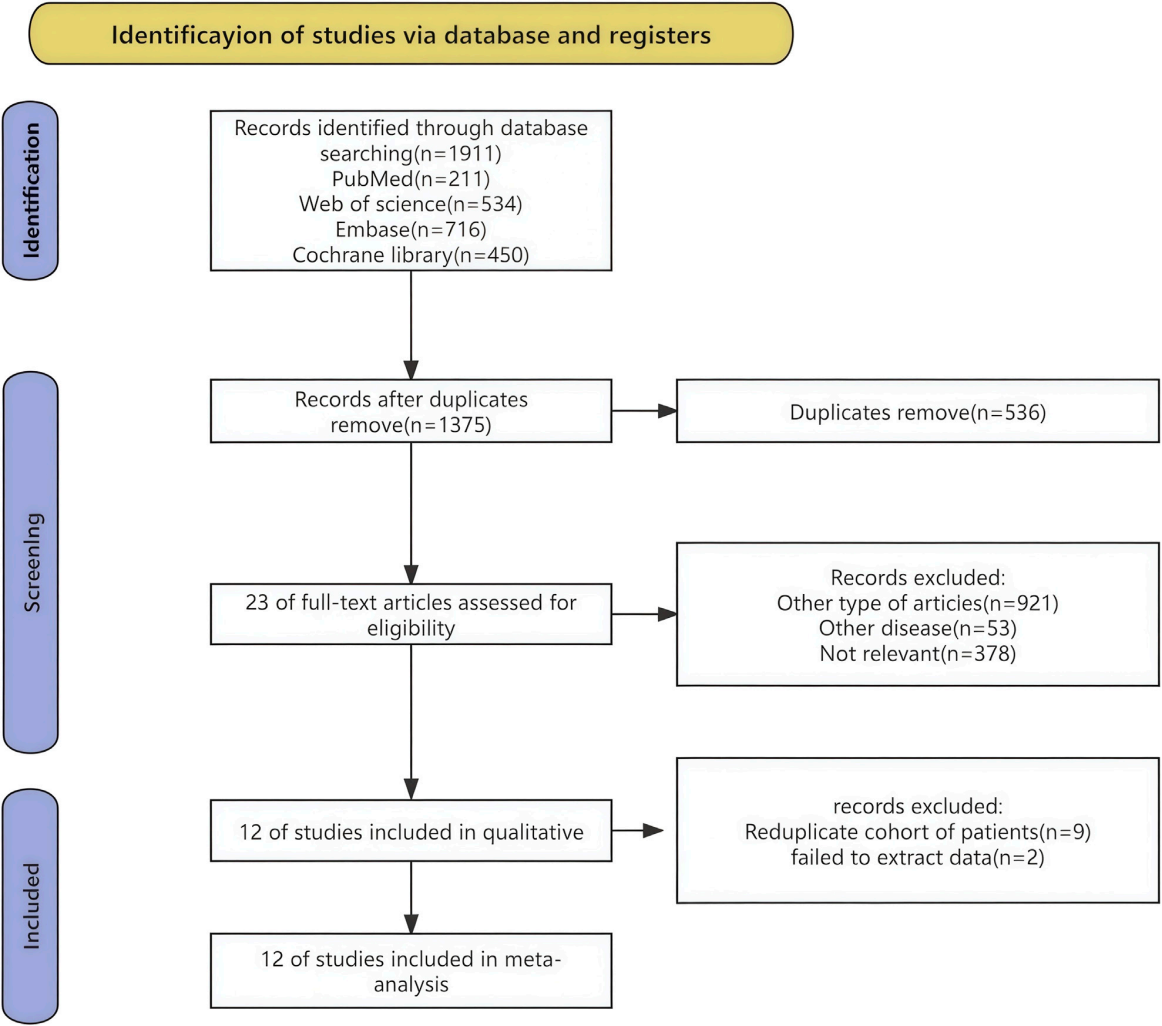
Flow chart of literature search strategies.

The procedure of selecting and integrating literature is depicted in Figure 1. A total of 1911 records were initially identified. Following the removal of superfluous research, a grand total of 1,375 papers remained. Based on the evaluation of the titles and abstracts, a total of 1,363 publications were considered unsuitable and so eliminated. A comprehensive review of the whole text led to the selection of 12 studies for included in this meta-analysis.

### 3.2 Patient characteristics

This meta-analysis comprised 12 RCTs (Zhang et al., 2020; Zhang et al., 2023; Xu et al., 2022; Slamon et al., 2024; Neven et al., 2023; Lu et al., 2022; Hortobagyi et al., 2022; Goetz et al., 2024; Finn et al., 2020; Albanell et al., 2022; Kalinsky et al., 2025; Noguchi et al., 2024) published between 2014 and 2025. These trials included a total of 4,957 patients who were diagnosed with untreated metastatic or advanced HR+/HER2-breast cancer. Among them, 2,877 patients were administered First-line CDK4/6 inhibitors together with ET, while 2080 patients received first-line ET alone. The registration ID, authors, year, subgroups, regimens, patients, age, and median follow-up of the included literature are shown in Table 1.

**TABLE 1:** Patient characteristics of included studies and patients.

Trial identifier	Study area	Trial phase	Author, year	Subgroups	Mean age (year)	Number of patients	Regimens	Receptor status	ECOG PS (%)			Median follow-up time (m)
								ER+	0	1	2	
FLIPPER	Spain and Ireland	Phase II trial	Albanell et al. (2022)	CDK4/6i + ET	64 (38–81)	94	P 125 mg/d plus fulvestrant 500 mg/28d; 36 m	94 (100%)	49 (52.1)	41 (43.6)	4 (4.3)	28.6
				ET	64 (42–82)	95	Placebo plus fulvestrant 500 mg/28d; 36 m	95 (100%)	53 (55.8)	40 (42.1)	2 (2.1)	
MONALEESA-2	Canada, Austria, et al.	Phase III trial	Hortobagyi et al. (2022)	CDK4/6i + ET	62 (23–91)	334	R 600 mg/d plus letrozole 2.5 mg/d; 88 m	N/A	205 (61.4)	129 (38.6)	0 (0)	79.2
				ET	63 (29–88)	334	Placebo plus letrozole 2.5 mg/d; 88 m	N/A	202 (60.5)	132 (39.5)	0 (0)	
MONALEESA-3	United States, South Korea, et al.	Phase III trial	Neven et al. (2023)	CDK4/6i + ET	63 (31–89)	237	R 600 mg/d plus fulvestrant 500 mg/d; 78 m	N/A	152 (64.1)	84 (35.4)	1 (0.4)	70.8
				ET	63 (34–86)	128	Placebo plus fulvestrant 500 mg/d; 78 m	N/A	83 (64.8)	44 (34.4)	1 (0.8)	
MONALEESA-7	United States, South Korea, et al.	Phase III trial	Lu et al. (2022)	CDK4/6i + ET	43 (25–58)	335	R 600 mg/d plus goserelin 3.6 mg/28d and NSAI (letrozole 2.5 mg/d or anastrozole 1 mg/d) or tamoxifen 20 mg/d; 66 m	331 (98.8%)	245 (73.1)	87 (26.0)	0 (0)	53.5
				ET	45 (29–58)	337	Placebo plus goserelin 3.6 mg/28d and NSAI (letrozole 2.5 mg/d or anastrozole 1 mg/d) or tamoxifen 20 mg/d; 66 m	335 (99.4%)	255 (75.7)	78 (23.1)	1 (0.3)	
PALOMA-1/TRIO-18	United States, South Korea, et al.	Phase II trial	Finn et al. (2020)	CDK4/6i + ET	63 (54–71)	84	P 125 mg/d plus letrozole 2.5 mg/d; 78 m	N/A	46 (54.8)	38 (45.2)	0 (0)	64.7
				ET	64 (56–70)	81	letrozole 2.5 mg/d; 78 m	N/A	45 (55.6)	36 (44.4)	0 (0)	
PALOMA-2	Germany, South Korea, et al.	Phase II trial	Slamon et al. (2024)	CDK4/6i + ET	62 (30–89)	444	P 125 mg/d plus letrozole 2.5 mg/d; 50 m	N/A	257 (57.9)	178 (40.1)	9 (2.0)	90.1
				ET	61 (28–88)	222	Placebo plus letrozole 2.5 mg/d; 50 m	N/A	102 (45.9)	117 (52.7)	3 (1.4)	
PALOMA-4	China, Singapore, et al.	Phase III trial	Xu et al. (2022)	CDK4/6i + ET	54 (31–70)	169	P 125 mg/d plus letrozole 2.5 mg/d; 62 m	N/A	84 (49.7)	85 (50.3)	N/A	52.8
				ET	54 (29–70)	171	Placebo plus letrozole 2.5 mg/d; 62 m	N/A	81 (47.4)	90 (52.6)	N/A	
DAWNA-2	China	Phase III trial	Zhang et al. (2023)	CDK4/6i + ET	54 (47–63)	303	D 150 mg/d plus letrozole 2.5 mg/d or anastrozole 1 mg/d; 34 m	N/A	141 (46.5)	161 (53.1)	1 (0.3)	21.6
				ET	57 (46–63)	153	Placebo plus letrozole 2.5 mg/d or anastrozole 1 mg/d; 34 m	N/A	69 (45.1)	84 (54.9)	0 (0)	
MONARCH plus	China, Brazil, et al.	Phase III trial	Zhang et al. (2020)	CDK4/6i + ET	54 (32–83)	311	150 mg/2 d plus NSAI (anastrozole 1 mg/d or letrozole 2.5 mg/d) or fulvestrant 500 mg/d; 28 m	N/A	N/A	N/A	N/A	16
				ET	54 (27–77)	152	Placebo plus NSAI (anastrozole 1 mg/d or letrozole 2.5 mg/d) or fulvestrant 500 mg/d; 28 m	N/A	N/A	N/A	N/A	
MONARCH 3	Japan, et al.	Phase III trial	Goetz et al. (2024)	CDK4/6i + ET	63 (38–87)	328	A 150 mg/2 d plus NSAI (anastrozole 1 mg/d or letrozole 2.5 mg/d); 98 m	N/A	192 (58.5)	136 (41.5)	0 (0)	97.2
				ET	63 (32–88)	165	Placebo plus NSAII (anastrozole 1 mg/d or letrozole 2.5 mg/d); 98 m	N/A	104 (63.0)	61 (37.0)	0 (0)	
post MONARCH	United States, et al.	Phase III trial	Kalinsky et al. (2025)	CDK4/6i + ET	58	182	abemaciclib 300 mg/d plus fulvestrant 500 mg/d	182 (100%)	104 (57.1)	78 (42.9)	0 (0)	13.0
				ET	61	186	Placebo 300 mg/d plus fulvestrant 500 mg/d	184 (98.9%)	107 (57.5)	79 (42.5)	0 (0)	
PATHWAY	Japan, et al.	Phase III trial	Noguchi et al. (2024)	CDK4/6i + ET	N/A	56	Palbociclib plus tamoxifen	N/A	N/A	N/A	N/A	40.9
				ET	N/A	56	Placebo plus tamoxifen	N/A	N/A	N/A	N/A	

CDK4/6i:CDK4/6 inhibitor, P:Palbociclib, R:Ribociclib D:Dalpiciclib, A:Abemaciclib

d, days; m, months.

ET, endocrine therapy.

NSAI, Non-Steroidal Aromatase Inhibitors

ECOG PS (Eastern Cooperative Oncology Group Performance Status) means the performance status score of the Eastern Cooperative Oncology Group in medicine.

ECOG PS represents the patient’s physical condition as a percentage, as follows:

Score 0: Completely normal activity ability, 100%.

Score 1: Able to walk freely and engage in light physical activities such as general housework or office work. The physical condition is about 90%–100%.

Score 2: Able to walk freely and take care of oneself, but has lost the ability to work. More than half of the day can get out of bed and move around. The physical condition is about 70%–80%.

N/A, Not Available.

### 3.3 Risk of bias

**FIGURE 2:**
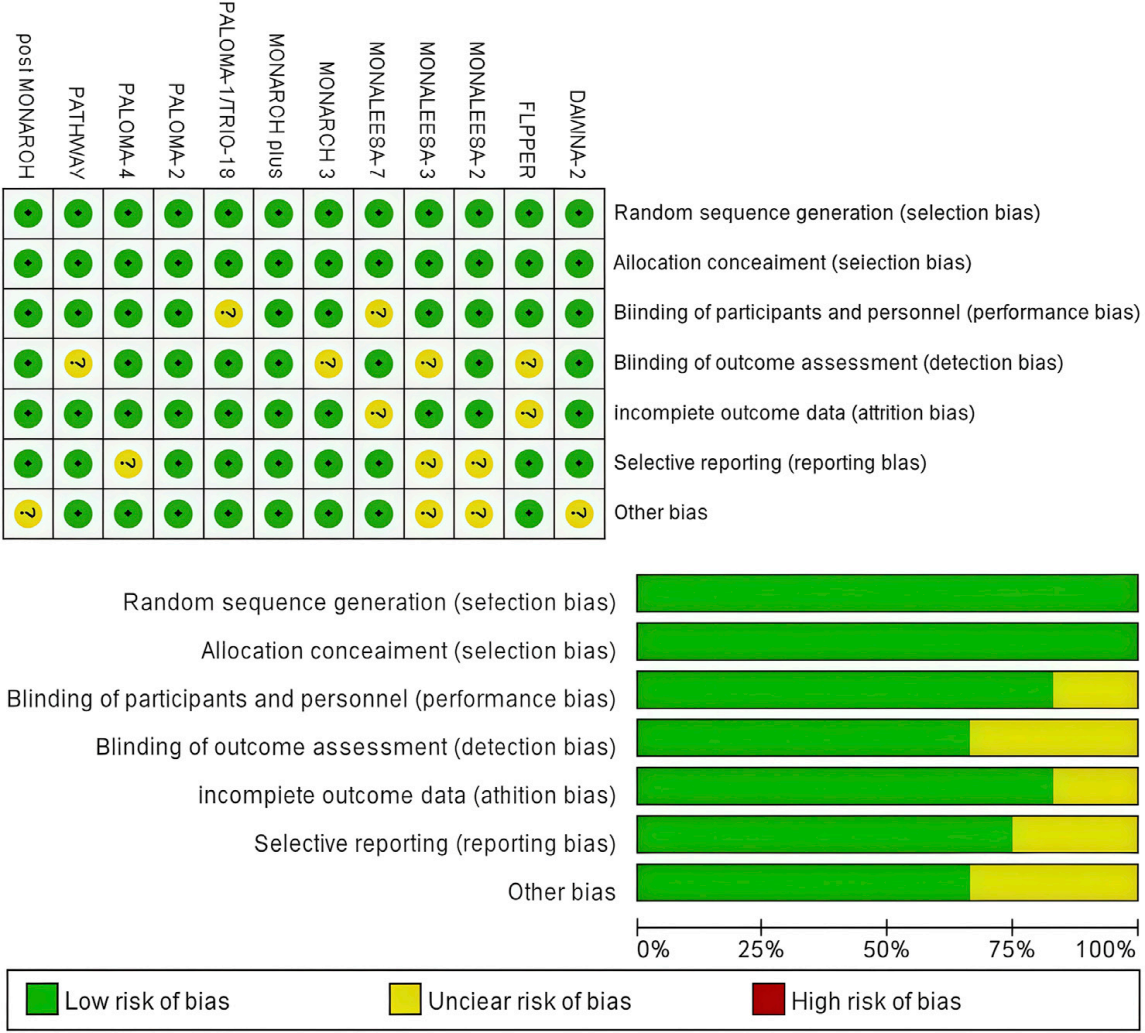
Risk of bias assessment diagram.

Figure 2 presents a concise overview of the risk of bias assessment results. Twelve studies produced a sufficient random sequence, twelve studies reported appropriate allocation concealment, ten studies clearly implemented participant blinding, eight studies reported outcome assessor blinding, ten studies provided complete outcome data, nine studies did not engage in selective reporting, and eight studies did not exhibit any other bias.

### 3.4 Efficacy outcomes

#### 3.4.1 ORR

**FIGURE 3:**
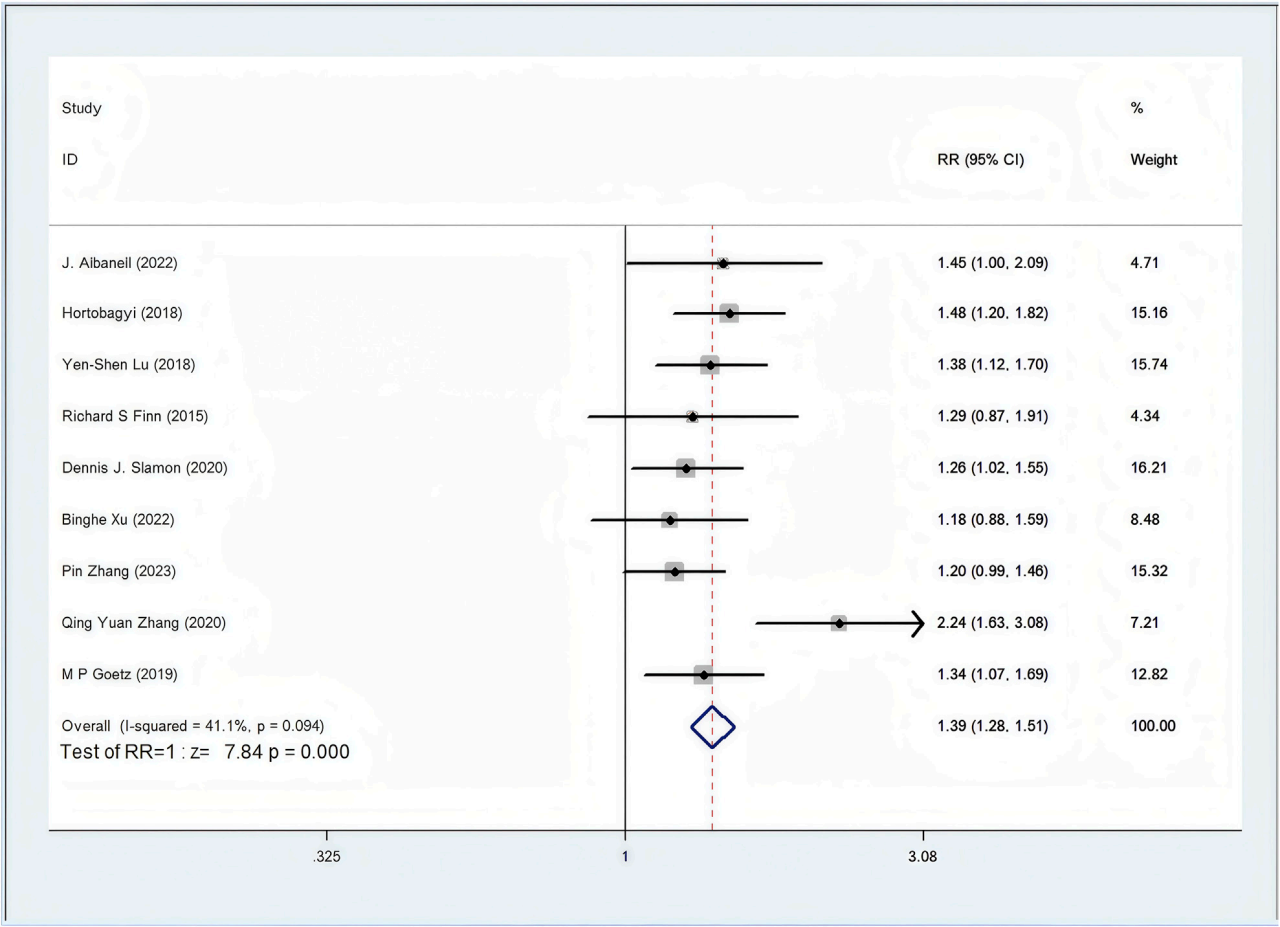
Forest plot of the meta-analysis for ORR.

Among the 12 RCTs considered, nine studies provided data regarding ORR (Zhang et al., 2020; Zhang et al., 2023; Xu et al., 2022; Slamon et al., 2024; Lu et al., 2022; Hortobagyi et al., 2022; Goetz et al., 2024; Finn et al., 2020; Albanell et al., 2022). The ORR of patients in the CDK4/6 inhibitors plus ET group was significant higher than that of the ET group (46.1% vs. 32.3%, RR = 1.39, 95% CI, 1.28 to 1.51, P < 0.01) (Figure 3). Sensitive analysis showed that the outcomes were stable (Supplementary Figure S1). In addition, we used funnel plot, Begg’s and Egger’s method to evaluate the publication bias regarding ORR. The results did not reveal any significant evidence of publication bias (Supplementary Figures S2-5).

#### 3.4.2 DCR

**FIGURE 4:**
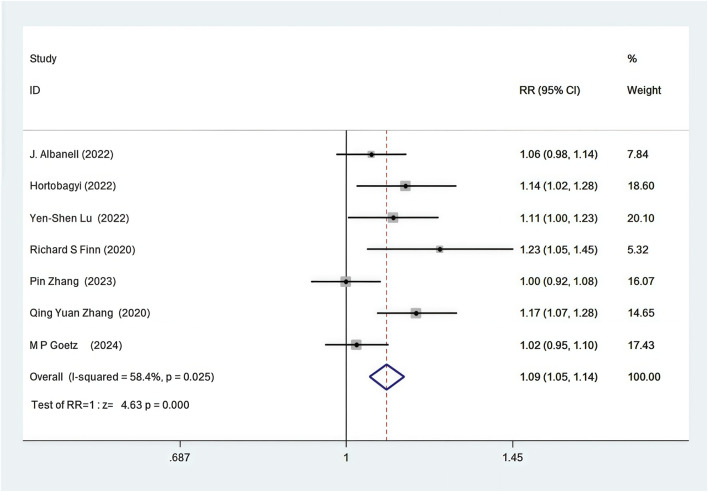
Forest plot of the meta-analysis for DCR.

Seven out of the 12 RCTs included in the analysis provided data on the disease control rate (DCR) (Zhang et al., 2020; Zhang et al., 2023; Lu et al., 2022; Hortobagyi et al., 2022; Goetz et al., 2024; Finn et al., 2020; Albanell et al., 2022). First-line CDK4/6 inhibitors combined with ET resulted in improved DCR compared to ET alone (82.5% vs. 72.9%, RR = 1.09, 95% CI, 1.05 to 1.14, P < 0.01) (Figure 4). Sensitive analysis showed that the outcomes were stable (Supplementary Figure S6). In addition, we used funnel plot, Begg’s and Egger’s method to evaluate the publication bias regarding DCR. The results did not reveal any significant evidence of publication bias (Supplementary Figures S7-10).

#### 3.4.3 PFS

**FIGURE 5:**
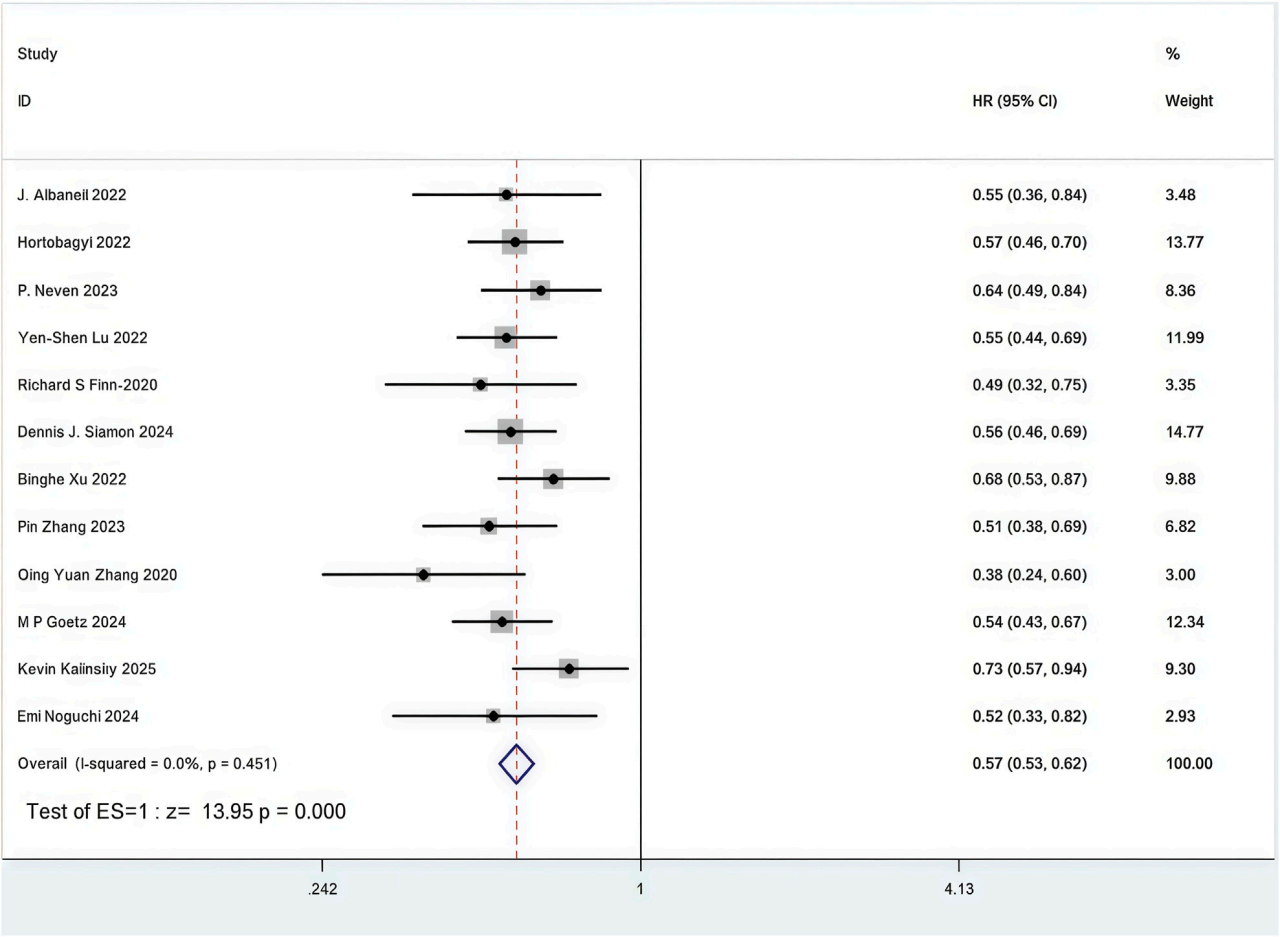
Forest plot of the meta-analysis for PFS.

All 12 RCTs included in the analysis presented Kaplan-Meier curves and hazard ratios (HR) for PFS (Zhang et al., 2020; Zhang et al., 2023; Xu et al., 2022; Slamon et al., 2024; Neven et al., 2023; Lu et al., 2022; Hortobagyi et al., 2022; Goetz et al., 2024; Finn et al., 2020; Albanell et al., 2022; Kalinsky et al., 2025; Noguchi et al., 2024). Combined use of First-line CDK4/6 inhibitors and ET was superior to ET in terms of PFS (HR: 0.57, 95%CI 0.53 to 0.62, P < 0.01) (Figure 5). Sensitive analysis showed that the outcomes were stable (Supplementary Figure S11). In addition, we used funnel plot, Begg’s and Egger’s method to evaluate the publication bias regarding PFS. The results did not reveal any significant evidence of publication bias (Supplementary Figures S12-15).

**FIGURE 6:**
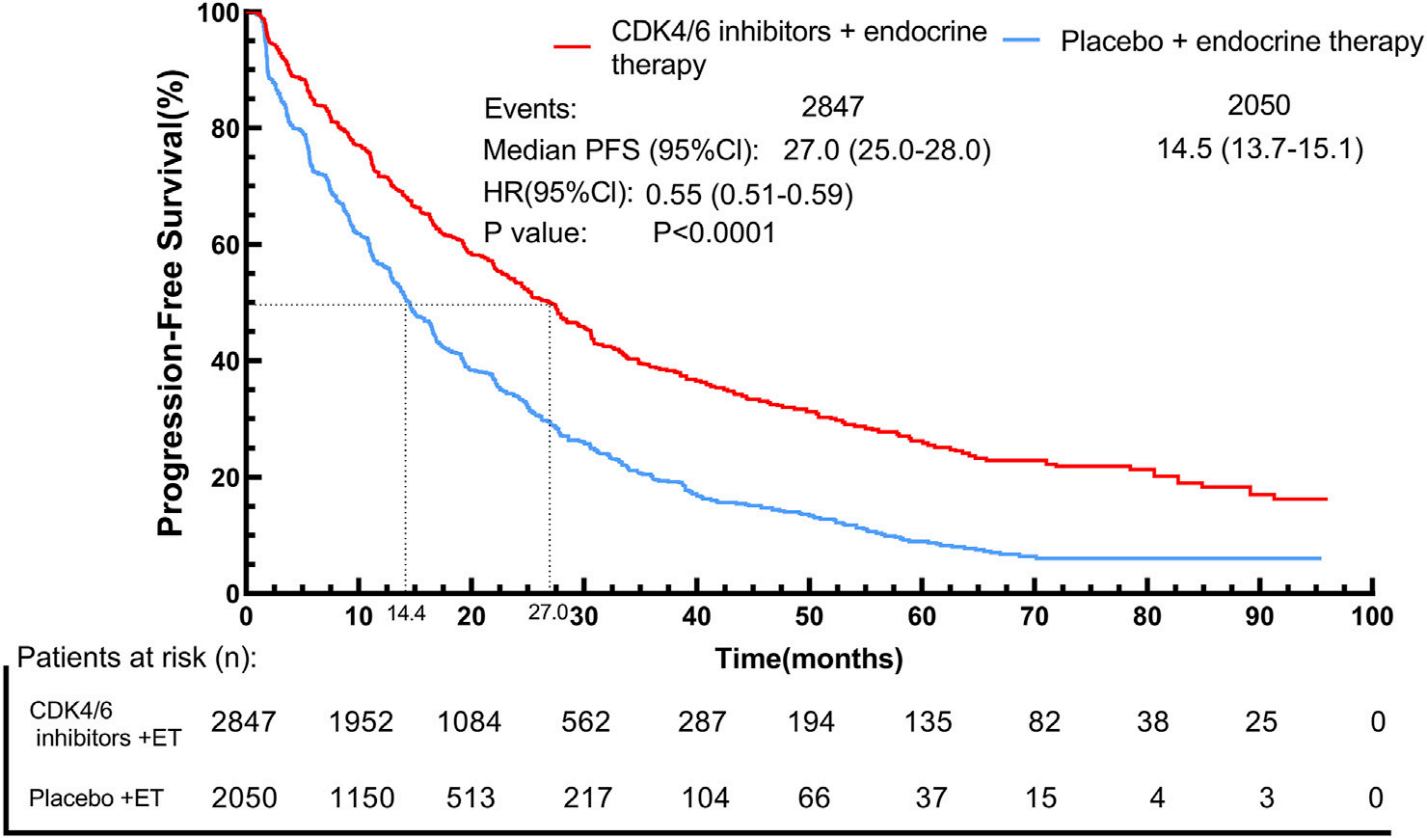
Kaplan-Meier curves for PFS.

By using the IPDformKM program, reconstruction of Kaplan-Meier curves for PFS provided a clear and comprehensible representation of oncological outcomes. Combined use of First-line CDK4/6 inhibitors and ET was superior to ET in terms of PFS (median survival time: 27.0 months versus 14.4 months, HR: 0.55, 95%CI 0.51 to 0.59, P < 0.01) (Figure 6).

#### 3.4.4 OS

**FIGURE 7:**
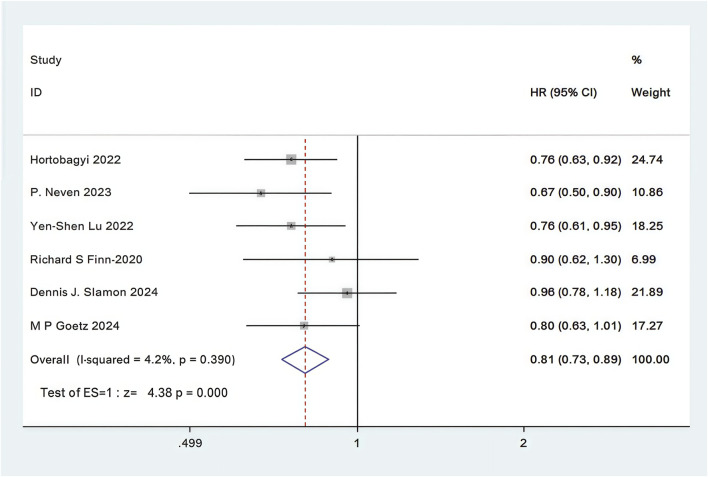
Forest plot of the meta-analysis for OS.

In all 12 RCTs, only six RCTs reported Kaplan-Meier curves and hazard ratios (HR) for OS (Slamon et al., 2024; Neven et al., 2023; Lu et al., 2022; Hortobagyi et al., 2022; Goetz et al., 2024; Finn et al., 2020). Combined use of First-line CDK4/6 inhibitors and ET was superior to ET in terms of OS (HR: 0.81, 95%CI 0.73 to 0.89, P < 0.01) (Figure 7). Sensitive analysis showed that the outcomes were stable (Supplementary Figure S16). In addition, we used funnel plot, Begg’s and Egger’s method to evaluate the publication bias regarding OS. The results did not reveal any significant evidence of publication bias (Supplementary Figures S17-20).

**FIGURE 8:**
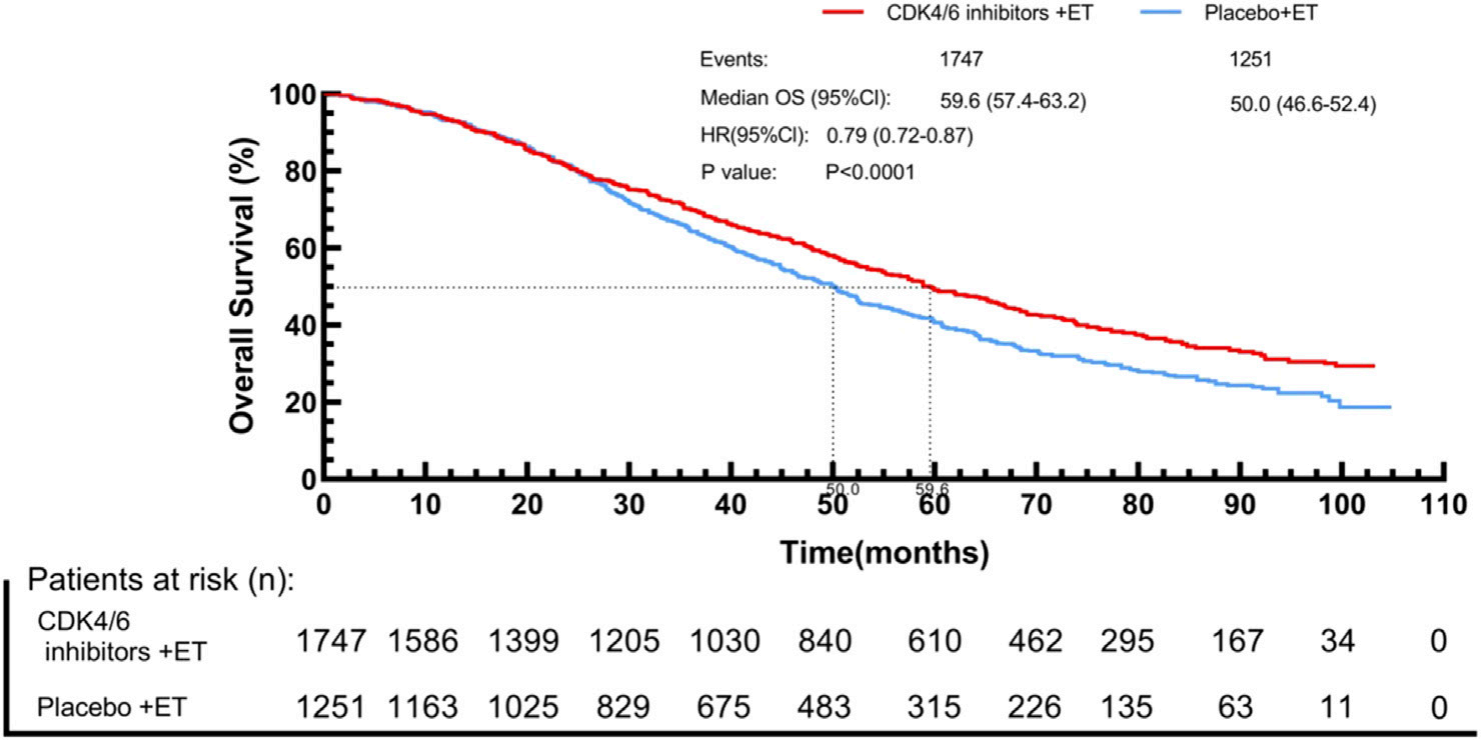
Kaplan-Meier curves for OS.

By using the IPDformKM program, reconstruction of Kaplan-Meier curves for OS provided a clear and comprehensible representation of oncological outcomes. Combined use of First-line CDK4/6 inhibitors and ET was superior to ET in terms of OS (median survival time: 59.6 months versus 50.0 months, HR: 0.79, 95%CI 0.72 to 0.87, P < 0.01) (Figure 8).

#### 3.4.5 Safety outcomes

**FIGURE 9:**
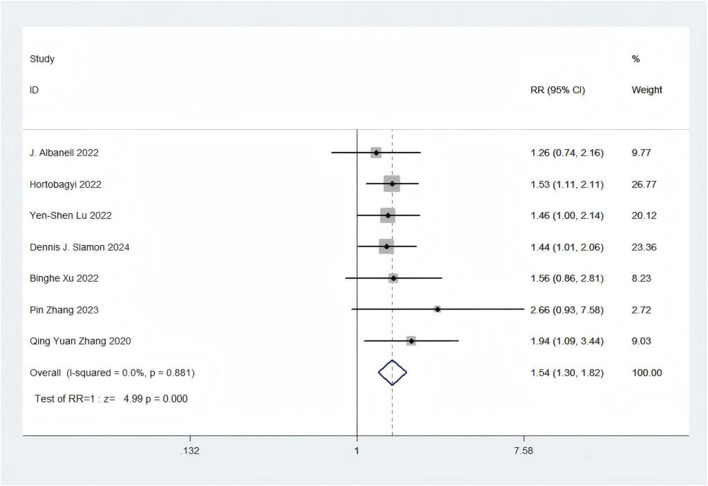
Forest plot of the meta-analysis for SAEs.

Eight trials presented data on SAEs (Zhang et al., 2020; Zhang et al., 2023; Xu et al., 2022; Slamon et al., 2024; Lu et al., 2022; Hortobagyi et al., 2022; Albanell et al., 2022; Kalinsky et al., 2025). With regards to safety, First-line CDK4/6 inhibitors plus ET exhibited a greater likelihood of encountering SAEs (RR = 1.54, 95% CI: 1.30 to 1.82, P < 0.01) (Figure 9) in comparison to ET. Sensitive analysis showed that the outcomes were stable (Supplementary Figure S21). In addition, we used funnel plot, Begg’s and Egger’s method to evaluate the publication bias regarding SAE. The results did not reveal any significant evidence of publication bias (Supplementary Figures S22-25).

### 3.5 Subgroup analysis regarding CDK4/6 inhibitors

Subgroup analysis regarding ORR, DCR, PFS and OS of different CDK4/6 inhibitors was performed (Table 2; Supplementary Figures S26-29). Regarding ORR and DCR, Palbociclib plus ET, Abemaciclib plus ET and Ribociclib plus ET all provided significant efficacy compared with ET, except for Dalpiciclib plus ET. Regarding PFS, four regimens all provided significant efficacy compared with ET. Regarding OS, Abemaciclib plus ET was significantly superior to ET, but Palbociclib plus ET or Ribociclib plus ET was not. The data of OS in the Dalpiciclib plus ET was not available.

**TABLE 2:** Results of Subgroup analysis for ORR, DCR, PFS and OS (CDK4/6 i + ET vs. ET).

	ORR				DCR				PFS				OS			
Group	No.of studies	RR (95%CI)	P	I^2^ (%)	No.of studies	RR (95%CI)	P	I^2^ (%)	No.of studies	HR (95%CI)	P	I^2^ (%)	No.of studies	HR (95%CI)	P	I^2^ (%)
Any CDK4/6 i + ET vs. ET																
Total	9	1.39 (1.28,1.51)	0.00	41	7	1.09 (1.05,1.14)	0.00	58	12	0.57 (0.53,0.62)	0.00	0	6	0.81 (0.73,0.89)	0.00	4
P + ET	4	1.27 (1.10,1.47)	0.00	0	2	1.13 (1.04,1.23)	0.00	74	5	0.58 (0.51,0.66)	0.00	0	2	0.95 (0.79,1.13)	0.54	0
R + ET	2	1.43 (1.23,1.66)	0.00	0	2	1.13 (1.04,1.21)	0.00	0	3	0.58 (0.51,0.66)	0.00	0	3	0.74 (0.65,0.85)	0.00	0
D + ET	1	1.20 (0.99,1.46)	0.06	-	1	1.00 (0.92,1.08)	0.97	-	1	0.51 (0.38,0.69)	0.00	-	-	-	-	-
A+ ET	2	1.67 (1.39,2.01)	0.00	85	2	1.09 (1.03,1.15)	0.00	82	3	0.58 (0.50,0.68)	0.00	47	1	0.80 (0.63,1.01)	0.06	-
Post-menopause vs. Mixed type																
Total	9	1.39 (1.28.1.51)	0.00	41	7	1.09 (1.05,1.14)	0.00	58	12	0.57 (0.53,0.62)	0.00	0	6	0.81 (0.73.0.89)	0.00	4
Post-menopause	7	1.43 (1.30.1.58)	0.00	48	5	1.29 (1.12.1.49)	0.00	80	8	0.57 (0.52,0.63)	0.00	0	5	0.82 (0.73.0.91)	0.00	19
Mixed type	2	1.29 (1.12.1.49)	0.00	0	2	1.08 (0.99.1.13)	0.08	88	4	0.59 (0.51,0.67)	0.00	0	1	0.78 (0.81.0.95)	0.02	-

Abbreviations: CI, confidence interval; ORR, objective response rate; DCR, disease control rate; PFS, Progression-free survival; OS, overall survival; i,inhibitors; ET, endocrine therapy; P,Palbociclib; R,Ribociclib; D,Dalpiciclib; A,Abemaciclib.

Subgroup analysis regarding ORR, DCR, PFS and OS of different menopausal status was performed (Table 2; Supplementary Figures S30-33). Patients in eight trials were post-menopause, while data of post-menopause and pre-menopause patients (mixed patients) could not be separated in four trials. For post-menopause patients, CDK4/6 inhibitors plus ET provided significant efficacy compared with ET in terms of ORR, DCR, PFS and OS. For mixed patients, CDK4/6 inhibitors plus ET provided significant efficacy compared with ET in terms of ORR and PFS, but not DCR or OS.

### 3.6 Evidence certainty

**FIGURE 10:**
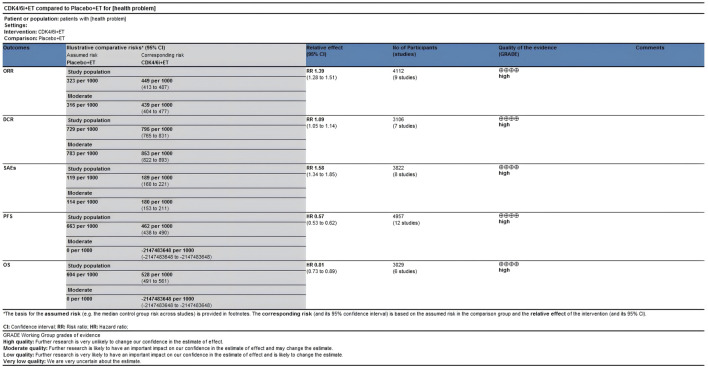
The quality of the evidence analysis (GRADE approach).

The certainty of evidence assessed for the various outcomes as per GRADE (Grading of Recommendations, Assessment, Development and Evaluations) criteria were of high certainty category (Figure 10).

## 4 Discussion

In the last 10 years, the most notable progress in treating HR-positive, HER2-negative advanced or metastatic breast cancer has been the inclusion of CDK4/6 inhibitors together with ET in global treatment recommendations. This holds true for both the initial treatment and the subsequent therapy after disease progression after ET (Finn et al., 2015; Pernas et al., 2018; Spring et al., 2019). Our meta-analysis compared the long-term effectiveness and safety of First-line CDK4/6 inhibitors plus endocrine therapy versus endocrine therapy in patients with HR+/HER2-metastatic or advanced breast cancer, and our results revealed that CDK4/6 inhibitors plus ET considerably improved ORR, DCR, PFS and OS, with acceptable safety.

Elaborate regulatory mechanisms are present in the normal cell cycle to guarantee precise completion of each cell cycle. The CDK4/6 complex is a crucial regulator of the cell cycle, functioning by its interaction with cyclin D (Nair et al., 2008). Estrogen stimulation in breast cancer triggers the activation of the ER signaling pathway, resulting in increased production of cyclin D and CDK4/6, and ultimately leading to unregulated cell growth (Altucci et al., 1997). Studies using mouse genetics have confirmed that cyclin D-CDK4/6 kinases play a crucial role in the development of several types of tumors, making them promising targets for therapy. Exploration of genetic and cell culture techniques revealed the reliance of breast cancer cells on CDK4/6 (Geum et al., 1997). Thus, due to the crucial regulatory function of CDK4/6 in the cell cycle, CDK4/6 inhibitors have been developed as anticancer medications (Sherr and Roberts, 1999). One of the fundamental biological features of malignant tumors is the abnormal proliferation and malignant transformation of tumor cells resulting from the disruption of cell cycle control. By specifically inhibiting cyclin-dependent kinases 4 and 6 (CDK4/6), CDK4/6 inhibitors reset the cell cycle and impede cell growth in several tumor cells, including breast cancer. CDK4/6 inhibitors have been shown to significantly enhance the prognosis of patients diagnosed with HR + HER2-breast cancer. Since its development two decades ago, CDK4/6 inhibitors have accomplished remarkable success in the treatment of breast cancer (O'Leary et al., 2016; Asghar et al., 2015). The results of our study indicate that the combination of CDK4/6 inhibitors and ET provides a substantial survival advantage compared to ET in patients with HR+/HER2− progressed or metastatic HR + HER-2 breast cancer. The observed result was linked to a rise in median PFS from 16.2 months to 27.0 months and median OS from 50.0 months to 59.6 months. This improvement may be ascribed to the superior ORR and DCR attained in patients who received CDK4/6 inhibitors plus ET compared to ET.

In addition to HR+/HER2− breast cancer, CDK4/6 inhibitors have been employed in the treatment of other subtypes of breast cancer. In the MonarcHER trial led by Sara M Tolaney, 273 women with advanced ER+/HER2+ were enlisted and given a treatment combination consisting of fulvestrant, abemaciclib, and trastuzumab. The participants were subsequently compared to those who were administered conventional chemotherapy in combination with trastuzumab. After a median follow-up period of 19.0 months, the experimental objective was successfully accomplished. Empirical data has demonstrated that the dual use of CDK4/6 inhibitors and conventional chemotherapy resulted in improved survival rates, with well tolerated adverse effects (Tolaney et al., 2020). In patients with stage II–III triple-negative breast cancer (TPBC) in the MUKDEN 01 Plus trial, a four-drug neoadjuvant regimen including trastuzumab plus pyrotinib, dalpiciclib, and letrozole resulted in a tumor progression-free survival rate of 58% and an overall response rate of 92%, with an acceptable safety profile observed overall (Huo et al., 2020). The study undertaken by Xiuzhi Zhu is the initial investigation into the claim that the concurrent use of olaparib and palbociclib has a synergistic impact on inhibiting the progression of BRCAmut triple-negative breast tumors (TNBCs). This discovery presents novel therapeutic opportunities for target triple-negative breast cancers (TNBCs), especially those that exhibit resistance to olaparib (Zhu et al., 2021).

Moreover, CDK4/6 inhibitors are also being extensively investigated in other types of cancer. Neoadjuvant pyrotinib and letrozole coupled with dalpiciclib were evaluated in the MUKDEN 01 study to determine their therapeutic efficacy and safety in treating testicular cancer. These findings indicate that this combinational therapy shown a considerable anti-tumor efficacy (Niu et al., 2022). The study conducted by Yang et al. shown that CDK4/6 inhibitors enhanced the susceptibility of acute myeloid leukemia cells to cytotoxic agents (Yang et al., 2015). The study conducted by Patnaik shown that CDK4/6 inhibitors attained disease control rates of 49% in a group of 68 patients diagnosed with non-small-cell lung cancer (Patnaik et al., 2016). ADkins’ research demonstrated a 39% objective response rate for CDK4/6 inhibitors in 62 patients with advanced head and neck squamous cell carcinoma (Adkins et al., 2019).

Among the trials included, there were four different CDK4/6 inhibitors, including of Abemaciclib, Palbociclib, Ribociclib and Dalpiciclib. Subgroup analysis of different CDK4/6 inhibitors was performed to explore the heterogeneity (Table 2). In terms of PFS, Palbociclib plus ET, Abemaciclib plus ET, Dalpiciclib plus ET and Ribociclib plus ET all provided significant efficacy compared with ET. The positive results reinforced the approval of Palbociclib, Abemaciclib and Ribociclib. However, though four regimens all provided efficacy compared with ET in terms of ORR, the difference was not statistical significant in the Dalpiciclib plus ET group. Similarly, though Palbociclib plus ET, Abemaciclib plus ET and Ribociclib plus ET all demonstrated OS benefit, there was no statistical significance in the Palbociclib plus ET group or the Abemaciclib plus ET group. One possible reason was that the statistical results might be difficult to demonstrate the statistical difference because of the limited sample size. Besides, the difference regarding ORR, DCR and OS might be linked to the specific molecular structure and mechanisms of action of the four drugs. Palbociclib, Ribociclib, and Abemaciclib are orally accessible, highly specific small molecule inhibitors of CDK4/6. Palbociclib exhibits selective action for the CDK4/cyclin D1 kinase, with an IC50 of 0.011 μmol/L, and demonstrates minimal or no activity against a comprehensive panel of 274 other protein kinases, encompassing other CDKs as well as several tyrosine and serine/threonine kinases (Fry et al., 2004). Ribociclib inhibits the CDK4/cyclin D1 and CDK6/cyclin D3 enzyme complexes with IC50 values of 0.01 and 0.039 μM in biochemical experiments, respectively, demonstrating significant selectivity for CDK4/6 over other cyclin-dependent kinases (Sobhani et al., 2019). The IC50 values for abemaciclib mesylate are 1.6 nM for CDK4, 2.0 nM for CDK6, and 180 nM for pRb phosphorylation inhibition. Palbociclib and ribociclib possess analogous structures refined for enhanced selectivity towards CDK4/6, whilst abemaciclib features a distinct chemical structure that facilitates the inhibition of additional kinases, notably CDK9, despite this not resulting in CDK9 inhibition in cellular models (Gelbert et al., 2014; De Luca et al., 2018). Palbociclib and abemaciclib shown superior efficacy in inhibiting the phosphorylation of serine 807 and serine 780 of RB, in comparison to Ribociclib, as assessed in several breast cancer cell lines. The combination with ER antagonists was most successful with palbociclib and ribociclib, but abemaciclib had notable single-agent action (Chen et al., 2016). Dalpiciclib is a new, highly selective small molecule inhibitor of CDK4/6, with similar potencies against CDK4 (IC50, 12.4 nM) and CDK6 (IC50, 9.9 nM). Dalpiciclib has exhibited anti-tumor efficacy in various *in vitro* and xenograft models, predominantly through Rb-dependent cytostasis (Long et al., 2019; Wang et al., 2017). *In vivo* xenografts demonstrated that dalpiciclib exhibited comparable or marginally superior anti-tumor efficacy relative to palbociclib, without eliciting significant toxicity. Furthermore, in HR-positive breast cancer cell lines and xenografts, dalpiciclib was able to surmount the acquired resistance to endocrine therapy (Long et al., 2019). Given that only a single RCT has documented the efficacy of dalpiciclib in a Chinese cohort and that there was an absence of data on overall survival, it is advisable to conduct additional RCTs with extended follow-up in various countries to further validate the efficacy of dalpiciclib.

Subgroup analysis regarding menopausal status was performed to explore the heterogeneity caused by menopausal status. For post-menopause patients, CDK4/6 inhibitors plus ET provided significant efficacy compared with ET in terms of ORR, DCR, PFS and OS. For mixed patients, CDK4/6 inhibitors plus ET provided significant efficacy compared with ET in terms of ORR and PFS, but not DCR or OS. This result indicated that menopausal status might influence the efficacy of CDK4/6 inhibitors plus ET. Unfortunately, data of pre-menopause patients was not available in this meta-analysis. In order to assess the efficacy of CDK4/6 inhibitors plus ET for pre-menopausal patients, well planned RCTs are needed to obtain more detailed outcome data about pre-menopausal patients.

In terms of safety, our findings indicated that the incidence of SAEs was higher in the CDK4/6 inhibitors plus ET group compared to the ET group (19.48% vs. 11.94%, RR = 1.58, 95% CI: 1.34 to 1.85, P < 0.01). The combination of treatment strategy with CDK4/6 inhibitors may result in an inevitable rise in the occurrence of adverse events. The most frequent adverse effects include neutropenia, leukopenia, thrombocytopenia, anemia, fatigue, hypertension, arthritis, back pain, respiratory infection, elevated AST, and increased ALT. The most prevalent immune-related adverse events were neutropenia, leucopenia, thrombocytopenia, and elevated ALT levels. Toxicities are often manageable with dose modification and supportive treatment. Hematological toxicity is prevalent among all four inhibitors; however, certain hematological adverse events (AEs) are more pronounced with palbociclib and ribociclib compared to abemaciclib. For instance, grade 3–4 neutropenia manifests in 66%, 60% and 52% of patients receiving palbociclib, ribociclib and dalpiciclib, respectively, whereas it occurs in only 22% of patients treated with abemaciclib (Piezzo et al., 2020). All CDK4/6 inhibitors are recognized for their significant role in the proliferation of hematological precursors, exhibiting a cytostatic effect on neutrophil precursors that induces cellular quiescence; this effect is promptly reversed upon cessation of the drug, which justifies the intermittent administration schedule of palbociclib and ribociclib. Palbociclib is taken orally with meals to enhance drug absorption, at a dosage of 125 mg per day on a 3/1 schedule (21 days on, 7 days off). If necessary, the dose may be lowered to 100 mg per day and subsequently to a final dose of 75 mg per day (FDA, 2019). Ribociclib is administered orally at a dosage of 600 mg per day following a 3/1 regimen (21 days on, 7 days off); dose reductions to 400 mg per day and 200 mg per day are permitted as the final dosage (FDA, 2017a). Abemaciclib is linked to a reduced incidence of hematological toxicity, exhibiting greater selectivity for CDK4. It can be administered continuously at a dosage of 200 mg twice day as monotherapy or at an initial dosage of 150 mg twice daily in conjunction with endocrine therapy. (FDA, 2017b). There are specific toxicities unique to the various CDK4/6 inhibitors. Ribociclib is linked to a significant incidence of hepatotoxicity and reversible QT interval prolongation, but abemaciclib is related with a greater occurrence of diarrhea, tiredness, venous thromboembolic events, and elevated blood creatinine levels (Hortobagyi et al., 2016; Goetz et al., 2017; Dickler et al., 2017; Spring et al., 2017).

Recently, there have been several trials of emerging therapies for HER2-positive and HR-positive metastatic breast cancer that are very interesting. A recent trial revealled that the combination of trastuzumab emtansine (T-DM1) with ET can significantly improve PFS and OS in patients with HER2-positive and HR-positive metastatic breast cancer (Kınıkoğlu et al., 2024). Triple therapy (CDK4/6 inhibitor, ET and PD-1 inhibitor) may be a potential treatment to improve the efficacy. The combination therapy (CDK4/6 inhibitors, endocrine therapy, and PD-1 inhibitors) may provide a promising treatment worth studying. Nonetheless, the latest trial conducted by Ana C Garrido-Castro demonstrated that ribociclib combined with spartalizumab and fulvestrant in metastatic hormone receptor-positive breast cancer exhibited poor efficacy and increased hepatotoxicity, hence hindering further development (Ana et al., 2025). The impact of combining CDK 4/6 inhibitors with chemotherapy has been investigated in preclinical research, yielding inconclusive findings (Piezzo et al., 2020). The combination of CDK4/6 inhibitors with paclitaxel enhanced cellular apoptosis in HR-positive and TNBC models (Choi and Yoo, 2012). The combination of palbociclib and carboplatin exhibited reduced antitumor efficacy relative to carboplatin alone, but only in Rb-competent mice (Roberts et al., 2012). However, this meta-analysis does not address the comparison of CDK4/6 inhibitors plus ET with other emerging treatments for HR+/HER2-metastatic or advanced breast cancer. It is advised to conducted RCTs comparing CDK4/6 inhibitors plus ET with other emerging treatments in the future.

The present study possesses several advantages. First,our study was an updated meta-analysis to compare the long-term effectiveness and safety of first-line CDK4/6 inhibitors plus ET versus ET in patients with HR+/HER2-metastatic or advanced breast cancer. Due to the short follow-up of the RCTs included, the previous meta-analyses (Lin et al., 2020; Li et al., 2021; Li et al., 2020; Desnoyers et al., 2020; Brandão et al., 2020) failed to reported long-term outcomes about first-line CDK4/6 inhibitors plus ET. During the past 4 years, several RCTs have updated their long-term outcome data such as PFS and OS of longer than 5 years (Slamon et al., 2024; Neven et al., 2023; Lu et al., 2022; Goetz et al., 2024). Hence, it is essential to perform a meta-analysis that compares the long-term effectiveness and safety of First-line CDK4/6 inhibitors plus ET versus ET in patients with HR+/HER2-metastatic or advanced breast cancer. In addition, the Kaplan-Meier curves for OS and PFS were reconstructed to provide a distinct and understandable depiction of the oncology outcomes.

However, our study is subject to various limitations. First, as is the case with any meta-analysis, the intrinsic variability across the studies included in terms of patient baseline characteristics, disease stage, and treatment regimens may have influenced the findings. The paper could elaborate on the differences in treatment regimens across the included studies. The types of CDK4/6 inhibitors and endocrine therapies, dosages, treatment durations and combinations might impact the meta-analysis outcomes. Second, our meta-analysis mostly comprised studies conducted on populations with distinct demographic characteristics in Europe, Asia, and America. Therefore, additional research is necessary to assess the efficacy of this treatment combination in other ethnic groups and geographic populations. Third, a significant limitation was the absence of specific data for premenopausal patients. The trials predominantly involved postmenopausal populations or aggregated groups without disaggregated data for premenopausal participants. The lack of specific data for pre-menopausal women restricts the generalizability of the findings, considering the potential biological and therapeutic differences between pre-menopausal and postmenopausal patients. In addition, though SAEs with CDK4/6 inhibitors plus ET were analyzed, there were still limitations in understanding the long-term safety profile, including the potential for under reporting of late-emerging side effects in the included trials or a lack of long-term follow-up data. Besides, another disadvantage was the absence of patient-reported outcomes (PROs) in the analyzed studies. PROs are crucial for understanding the impact of treatment on quality of life, which is particularly relevant for long-term treatments in metastatic cancer and should be assessed in the future clinical studies.

In conclusion, the present meta-analysis reported long-term effectiveness and safety of First-line CDK4/6 inhibitors plus ET versus ET in patients with HR+/HER2-metastatic or advanced breast cancer. CDK4/6 inhibitors plus ET was linked to enhanced ORR, DCR, PFS and OS among patients with HR+/HER2-metastatic or advanced breast cancer, compared with ET. In addition, the heightened efficacy of CDK4/6 inhibitors plus ET was accompanied by a rise in undesirable side effects.

## Data Availability

The datasets presented in this study can be found in online repositories. The names of the repository/repositories and accession number(s) can be found in the article/Supplementary Material.
