# Supplementary material for: Comparative long-term outcomes of first-line CDK4/6 inhibitors plus endocrine therapy versus endocrine therapy in patients with HR+/HER2-metastatic or advanced breast cancer: a meta-analysis
Source: Front Pharmacol. 2025 Jul 25;16:1600892. doi: 10.3389/fphar.2025.1600892 (PMC12331727; doi:10.3389/fphar.2025.1600892)

## Supplement Figure Legends

**Supplement Figure 1** Sensitive analysis for ORR

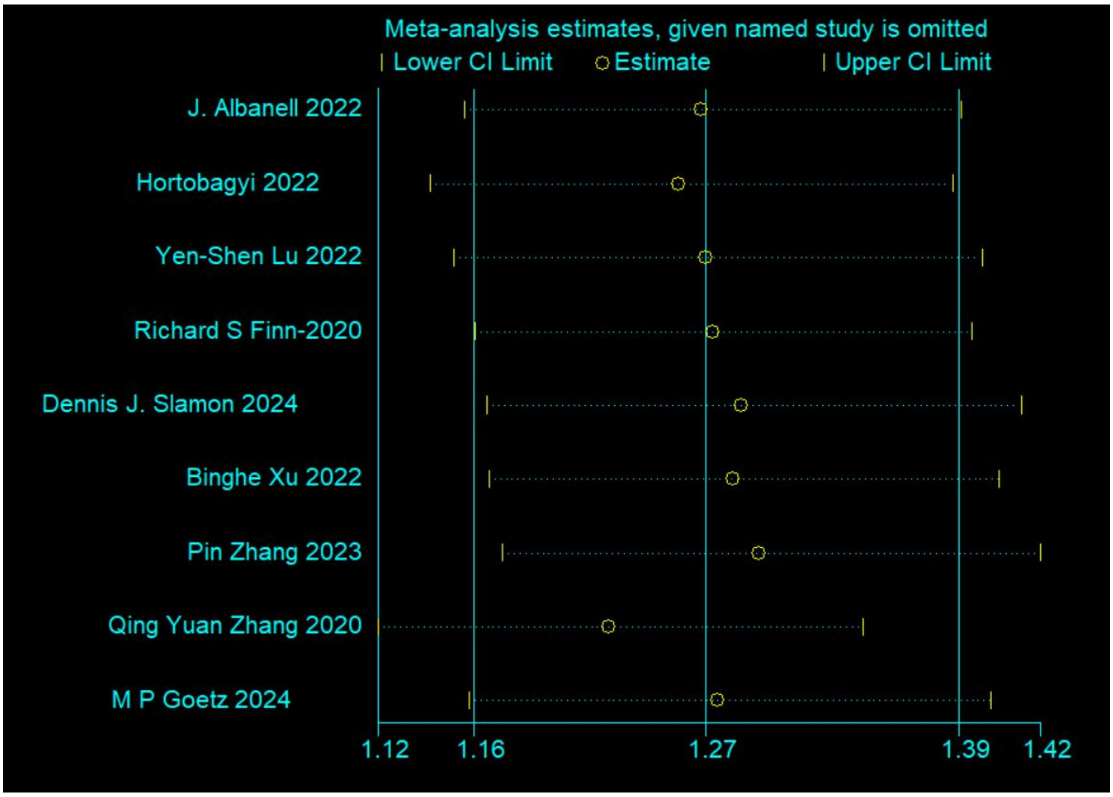

**Supplement Figure 2** funnel plot for ORR

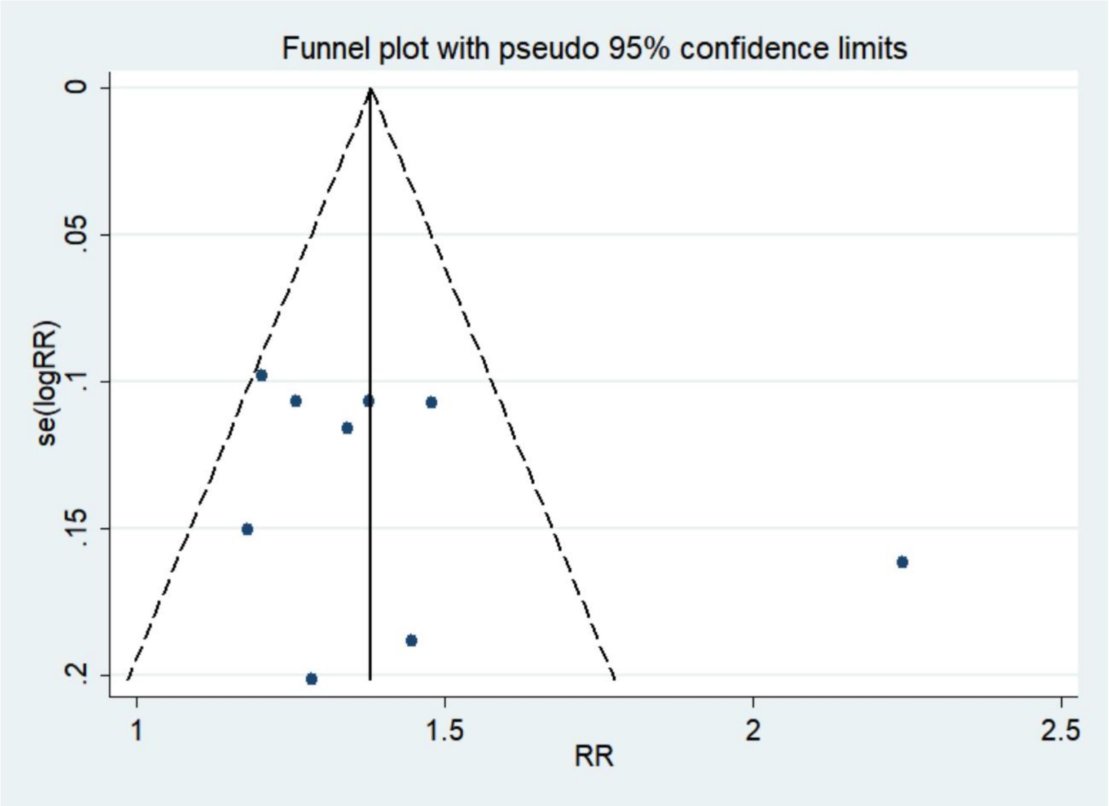


**Supplement Figure 3** Begg's for ORR

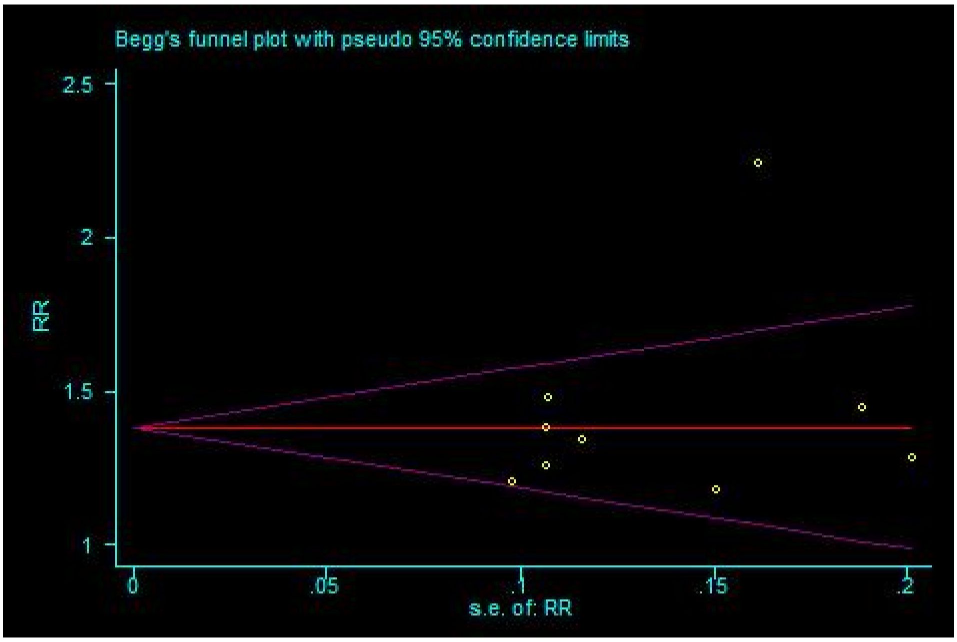

**Supplement Figure 4** Egger's for ORR

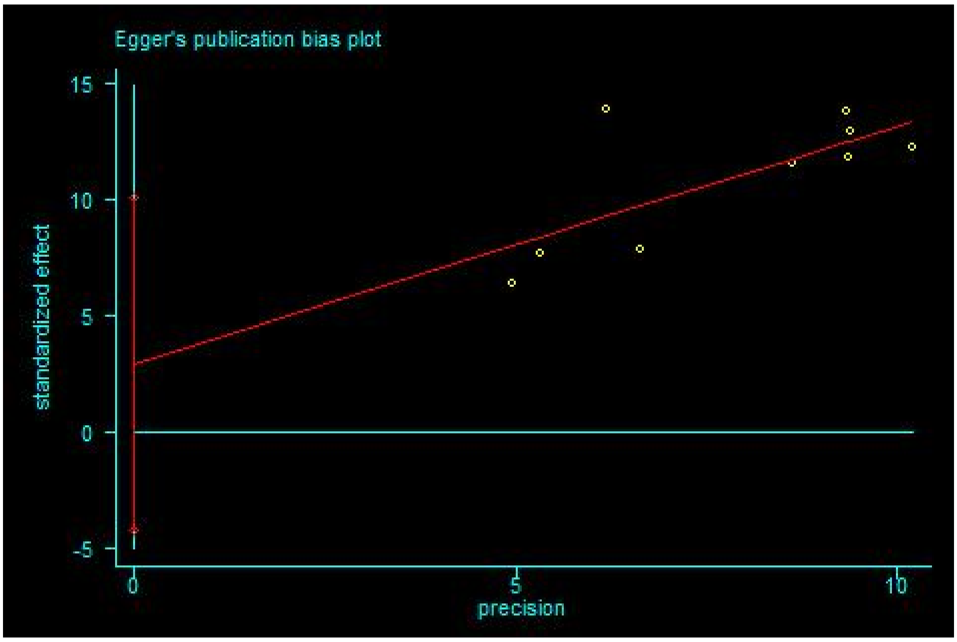


**Supplement Figure 5** Tests of publication bias for ORR

```
Tests for Publication Bias

Begg's Test

  adj. Kendall's Score (P-Q) =       8
          Std. Dev. of Score =    9.59
           Number of Studies =       9
                          z  =    0.83
                   Pr > |z|  =   0.404
                          z  =    0.73 (continuity corrected)
                   Pr > |z|  =   0.466 (continuity corrected)
```

Egger's test

| Std_Eff | Coef. | Std. Err. | t | P>\|t\| | [95% Conf. | Interval] |
|---|---|---|---|---|---|---|
| slope | 1.018458 | .3766368 | 2.70 | 0.030 | .1278533 | 1.909062 |
| bias | 2.990756 | 3.0163 | 0.99 | 0.354 | -4.141659 | 10.12317 |

**Supplement Figure 6** Sensitive analysis for DCR

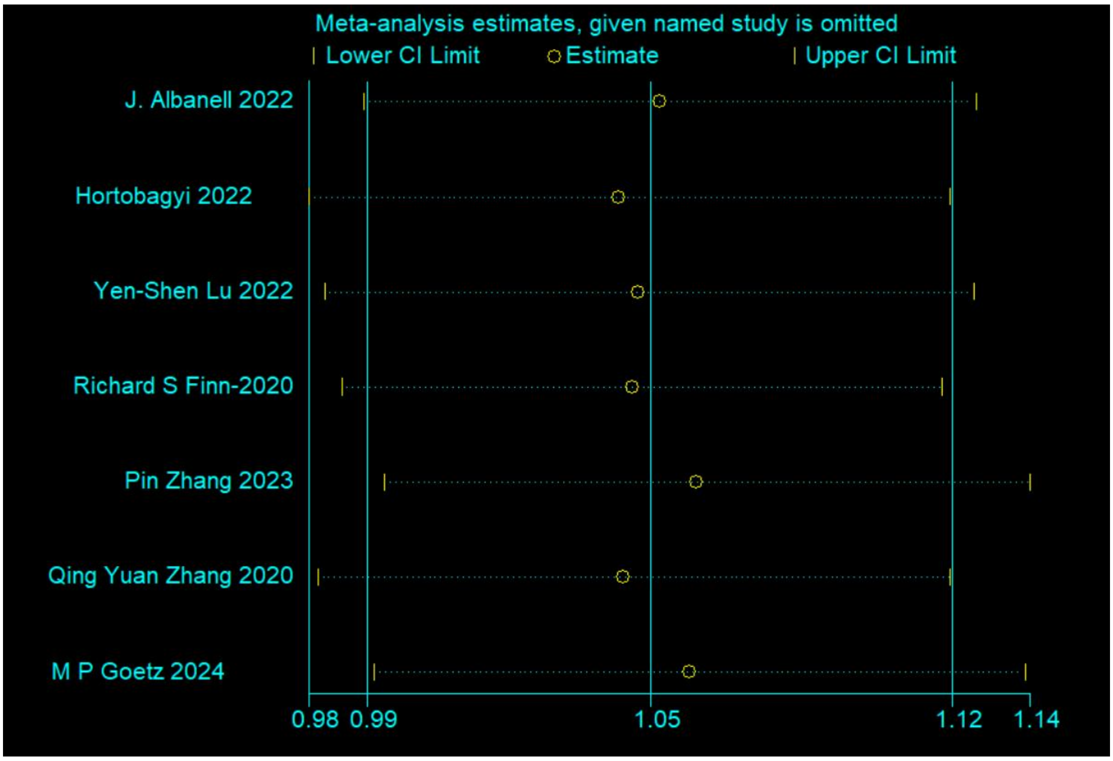


**Supplement Figure 7** funnel plot for DCR

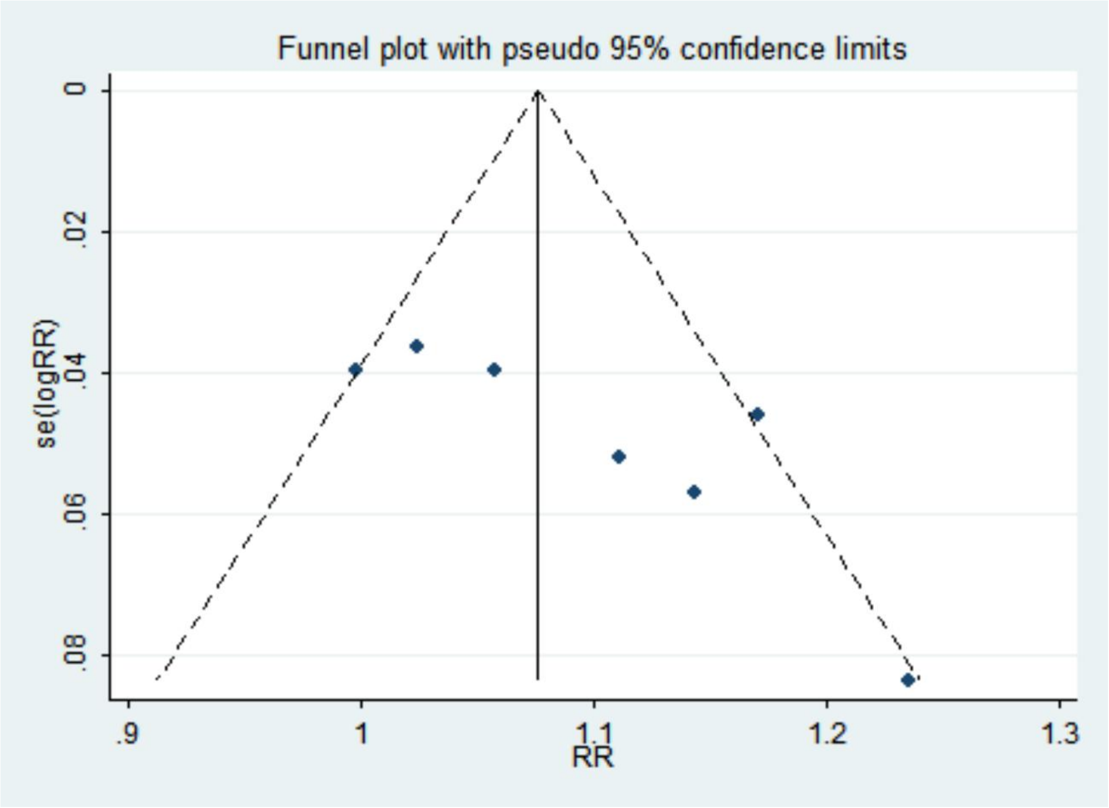

**Supplement Figure 8** Begg's for DCR

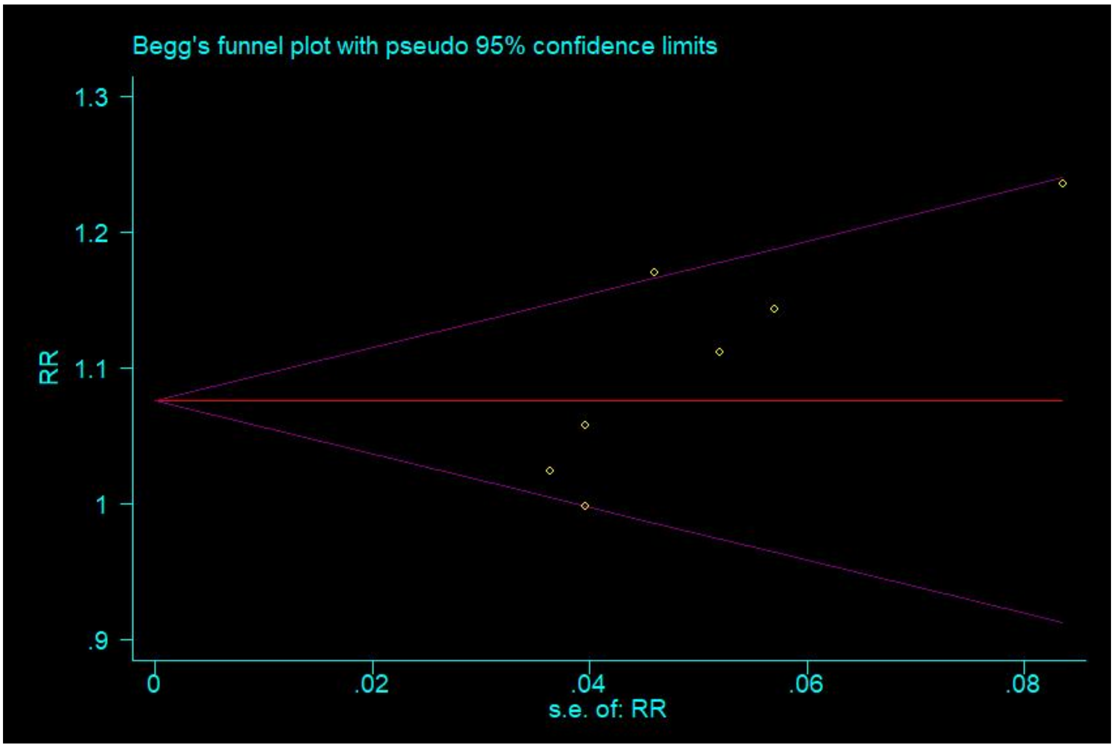


**Supplement Figure 9** Egger's for DCR

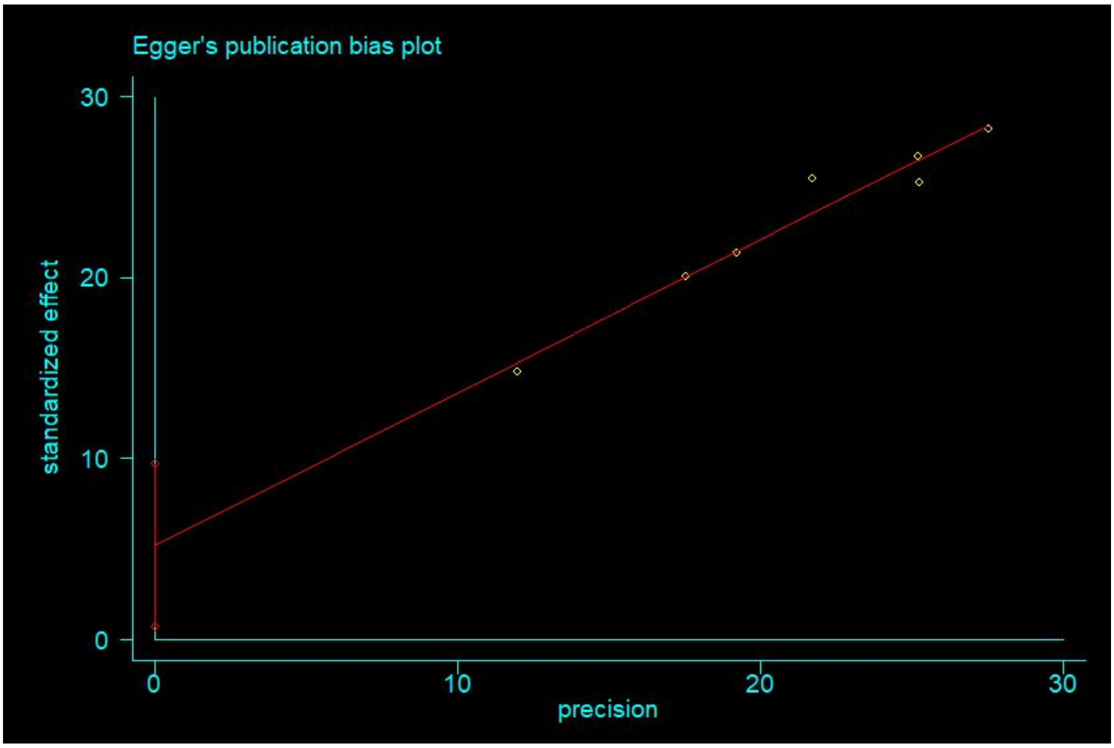

**Supplement Figure 10** Tests of publication bias for DCR

```
Tests for Publication Bias

Begg's Test

  adj. Kendall's Score (P-Q) =      13
          Std. Dev. of Score =    6.66
           Number of Studies =       7
                          z  =    1.95
                    Pr > |z| =   0.051
                          z  =    1.80 (continuity corrected)
                    Pr > |z| =   0.072 (continuity corrected)

Egger's test
```

| Std_Eff | Coef. | Std. Err. | t | P>\|t\| | [95% Conf. | Interval] |
|---|---|---|---|---|---|---|
| slope | .8422814 | .0804623 | 10.47 | 0.000 | .6354464 | 1.049116 |
| bias | 5.23273 | 1.752736 | 2.99 | 0.031 | .7271773 | 9.738282 |

**Supplement Figure 11** Sensitive analysis for hazard ratios (HR) of PFS

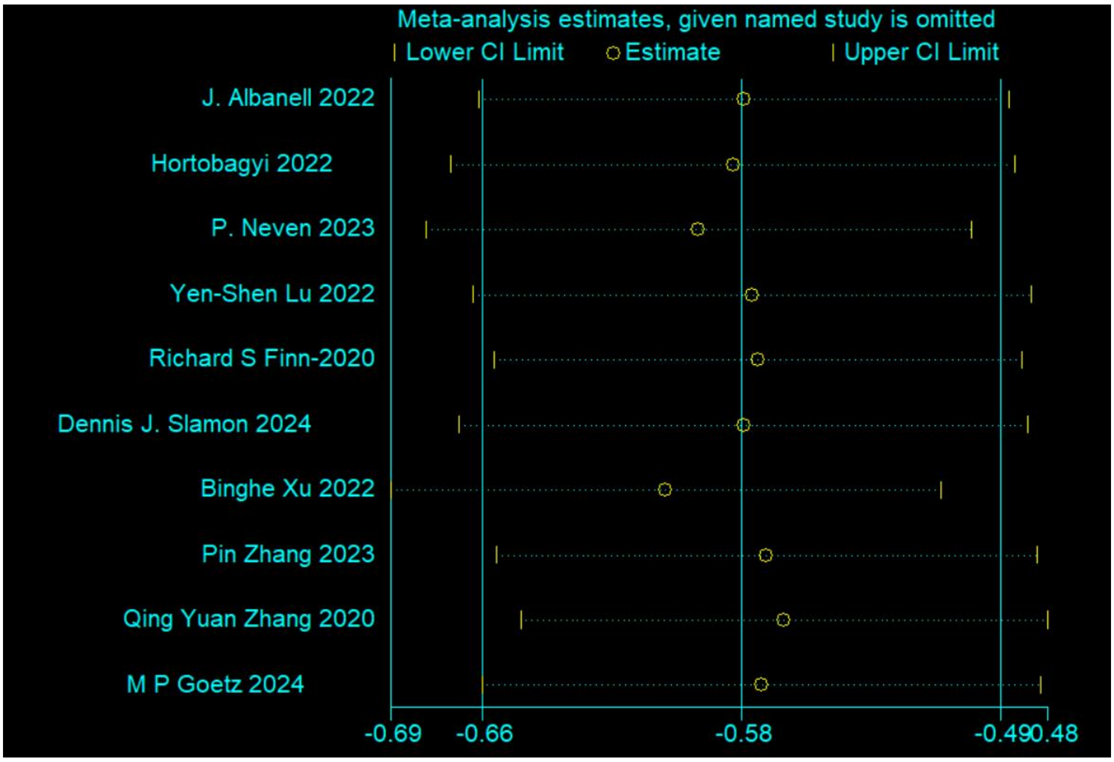

**Supplement Figure 12** funnel plot for PFS

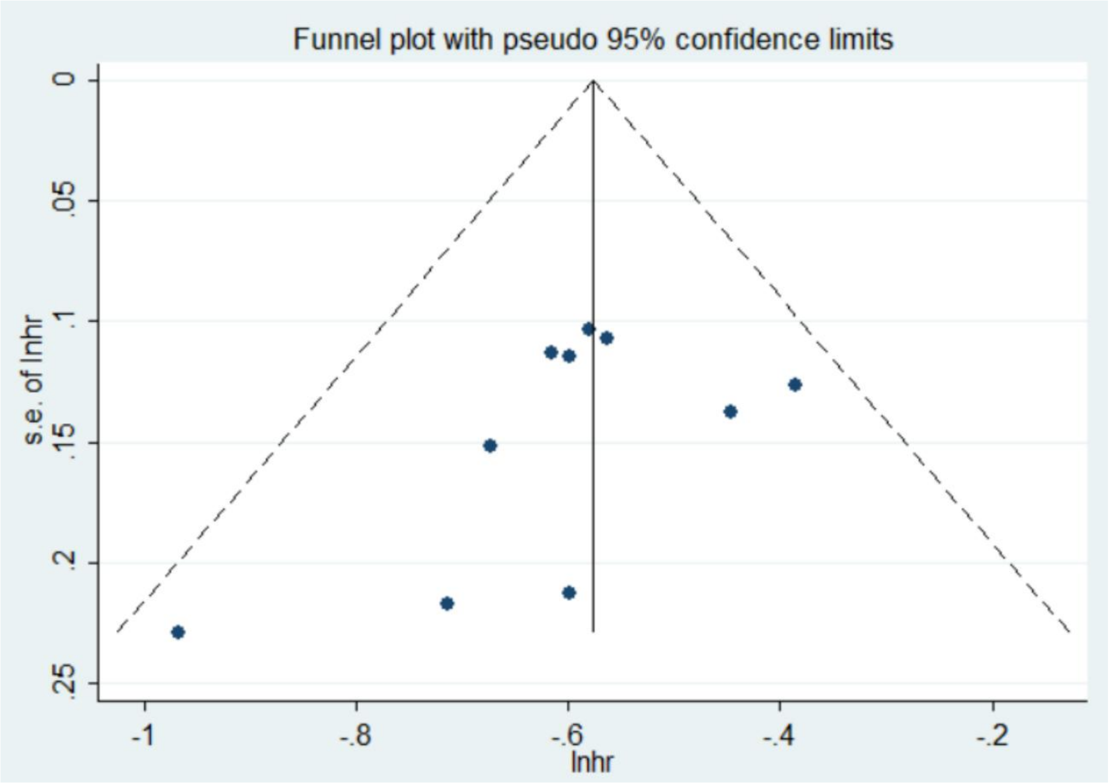


**Supplement Figure 13** Begg's for hazard ratios (HR) of PFS

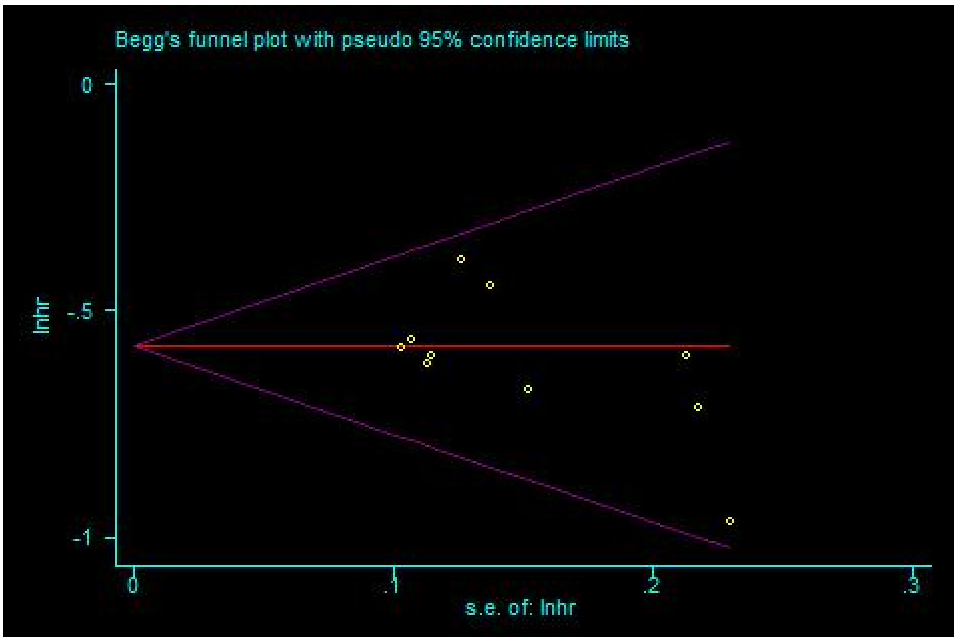

**Supplement Figure 14** Egger's for hazard ratios (HR) of PFS

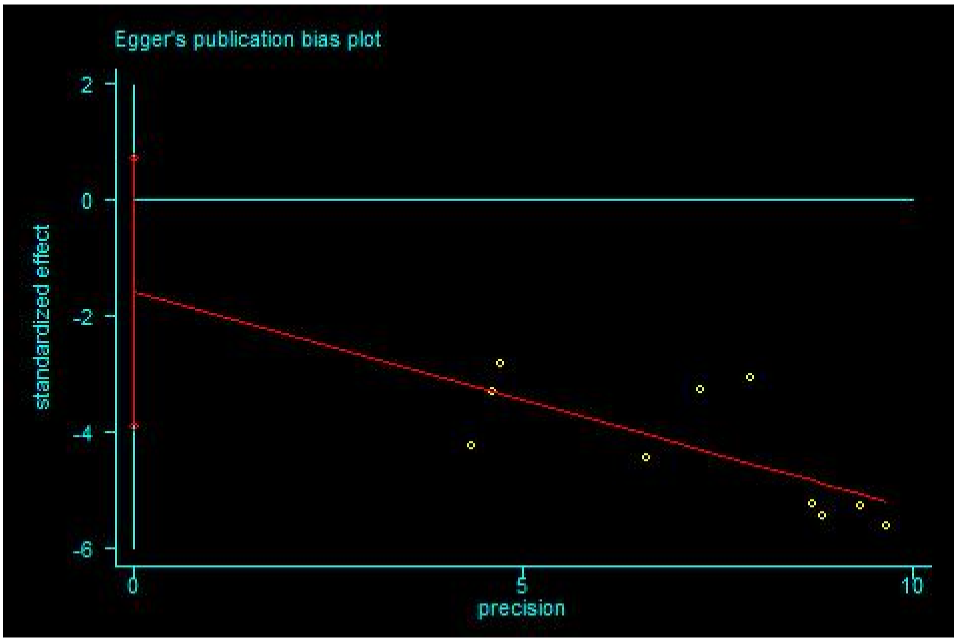


**Supplement Figure 15** Tests of publication bias for hazard ratios (HR) of PFS

```
Tests for Publication Bias

Begg's Test

  adj. Kendall's Score (P-Q) =     -17
          Std. Dev. of Score =   11.18
           Number of Studies =      10
                           z =   -1.52
                    Pr > |z| =   0.128
                           z =    1.43 (continuity corrected)
                    Pr > |z| =   0.152 (continuity corrected)

Egger's test
```

| Std_Eff | Coef. | Std. Err. | t | P>\|t\| | [95% Conf. | Interval] |
|---|---|---|---|---|---|---|
| slope | -.3741275 | .1341131 | -2.79 | 0.024 | -.6833929 | -.0648621 |
| bias | -1.561212 | .9995786 | -1.56 | 0.157 | -3.866244 | .7438209 |

**Supplement Figure 16** Sensitive analysis for hazard ratios (HR) of OS

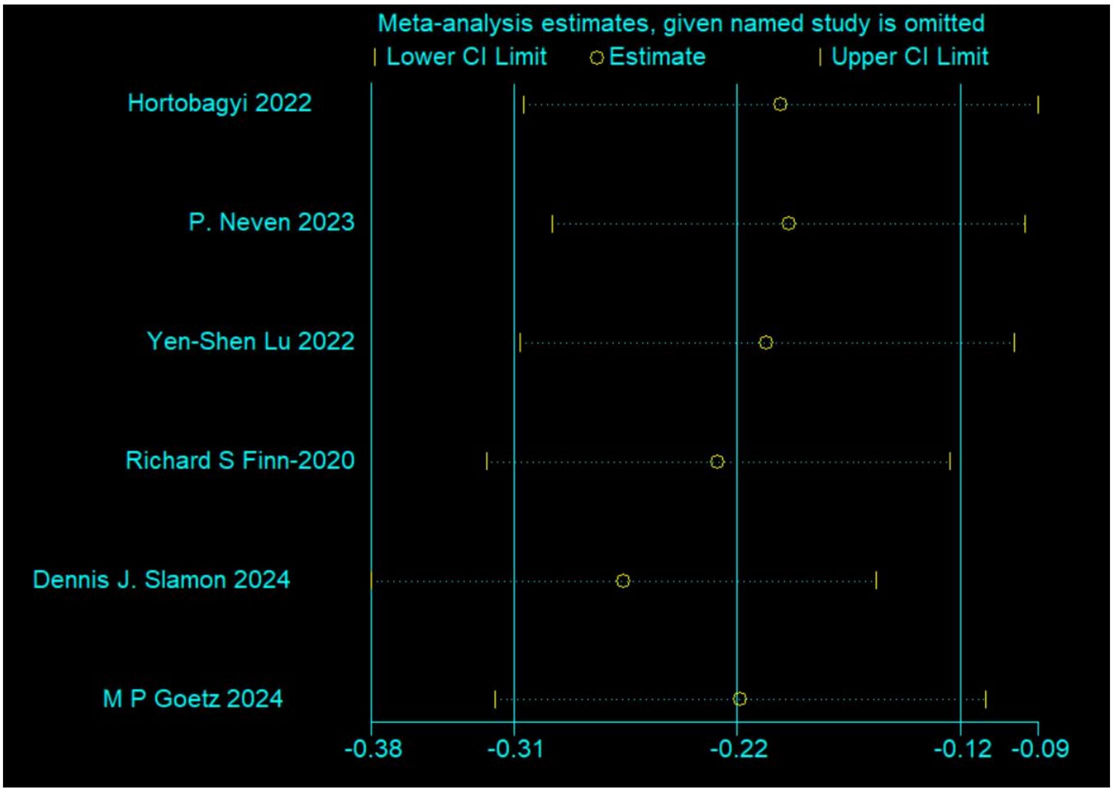


**Supplement Figure 17** funnel plot for OS

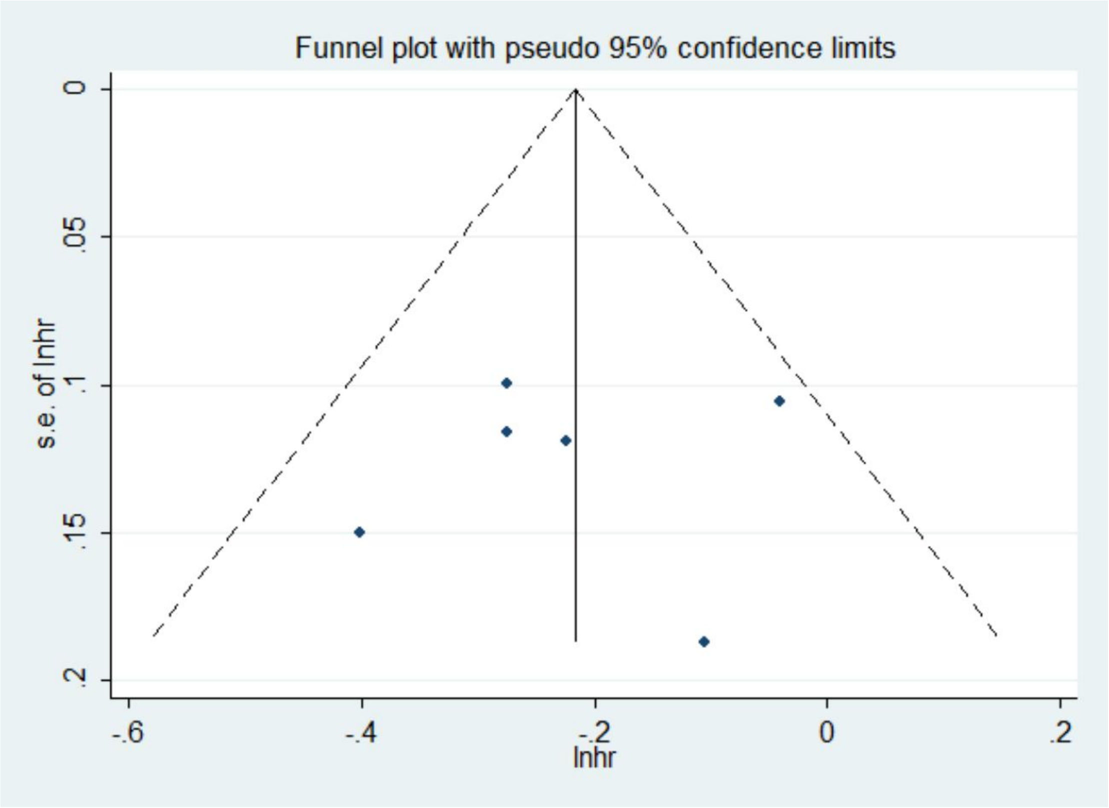

**Supplement Figure 18** Begg's for hazard ratios (HR) of OS

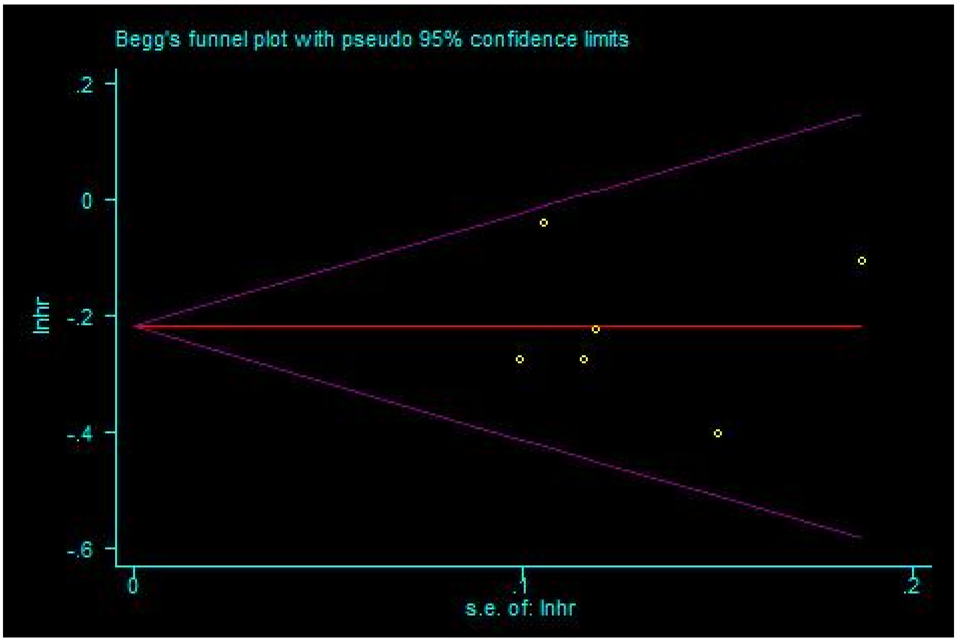


**Supplement Figure 19** Egger's for hazard ratios (HR) of OS

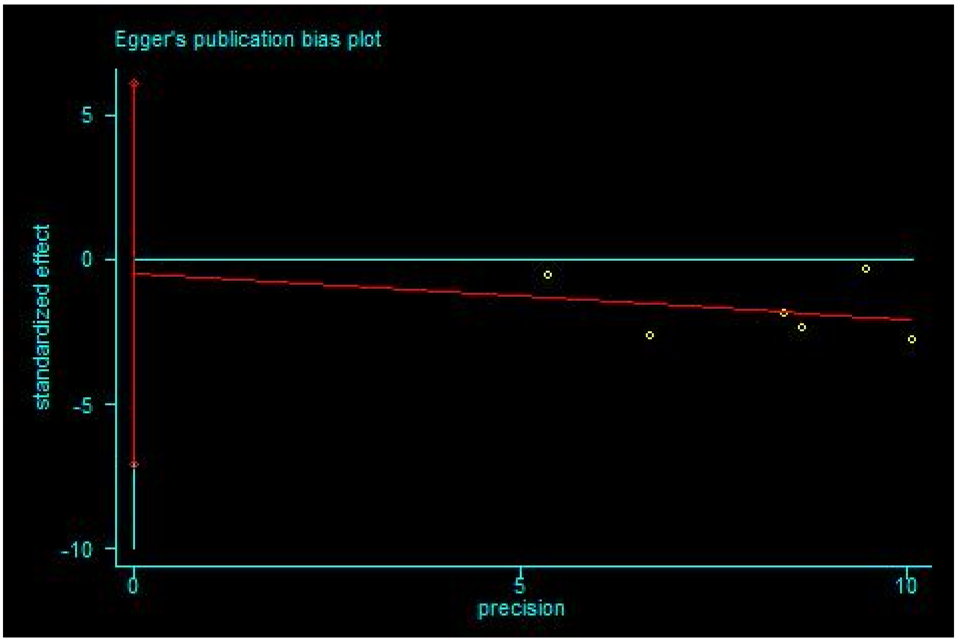

**Supplement Figure 20** Tests of publication bias for hazard ratios (HR) of OS

```
Tests for Publication Bias

Begg's Test

  adj. Kendall's Score (P-Q) =        1
          Std. Dev. of Score =     5.32
           Number of Studies =        6
                          z  =     0.19
                    Pr > |z| =    0.851
                          z  =     0.00 (continuity corrected)
                    Pr > |z| =    1.000 (continuity corrected)

Egger's test
```

| Std_Eff | Coef. | Std. Err. | t | P>\|t\| | [95% Conf. | Interval] |
|---|---|---|---|---|---|---|
| slope | -.1553427 | .2862889 | -0.54 | 0.616 | -.9502082 | .6395227 |
| bias | -.513569 | 2.365229 | -0.22 | 0.839 | -7.080497 | 6.053359 |

**Supplement Figure 21** Sensitive analysis for SAEs

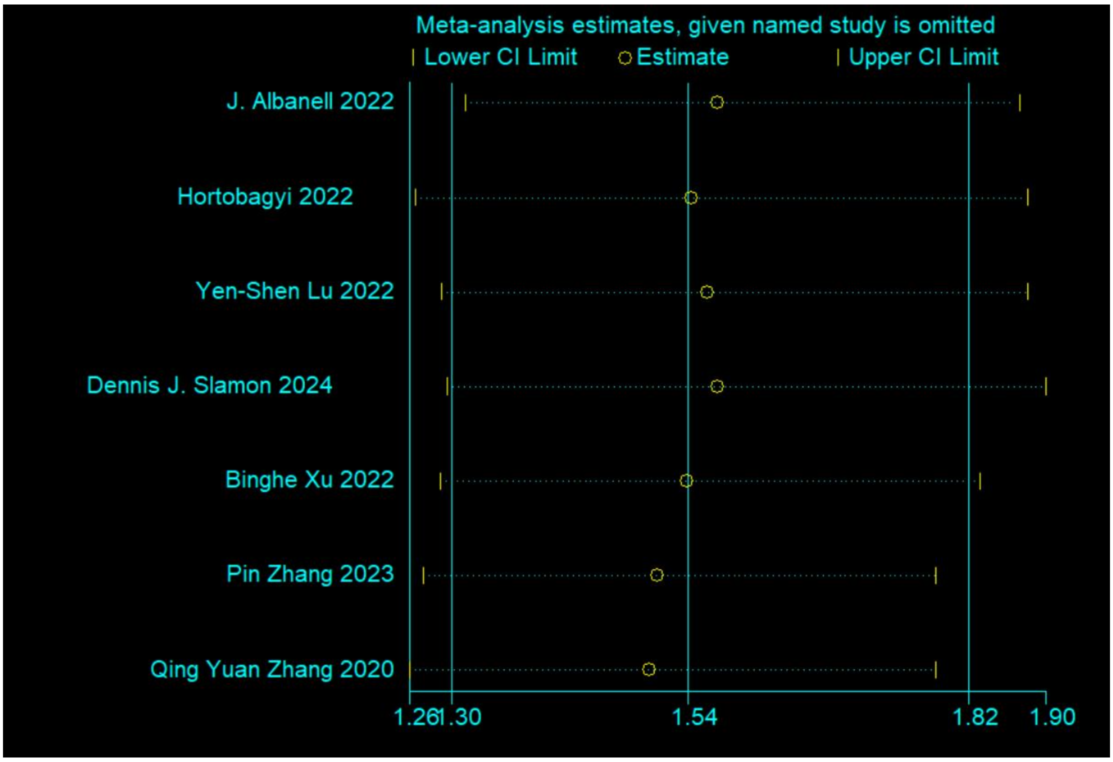

**Supplement Figure 22** funnel plot for SAEs

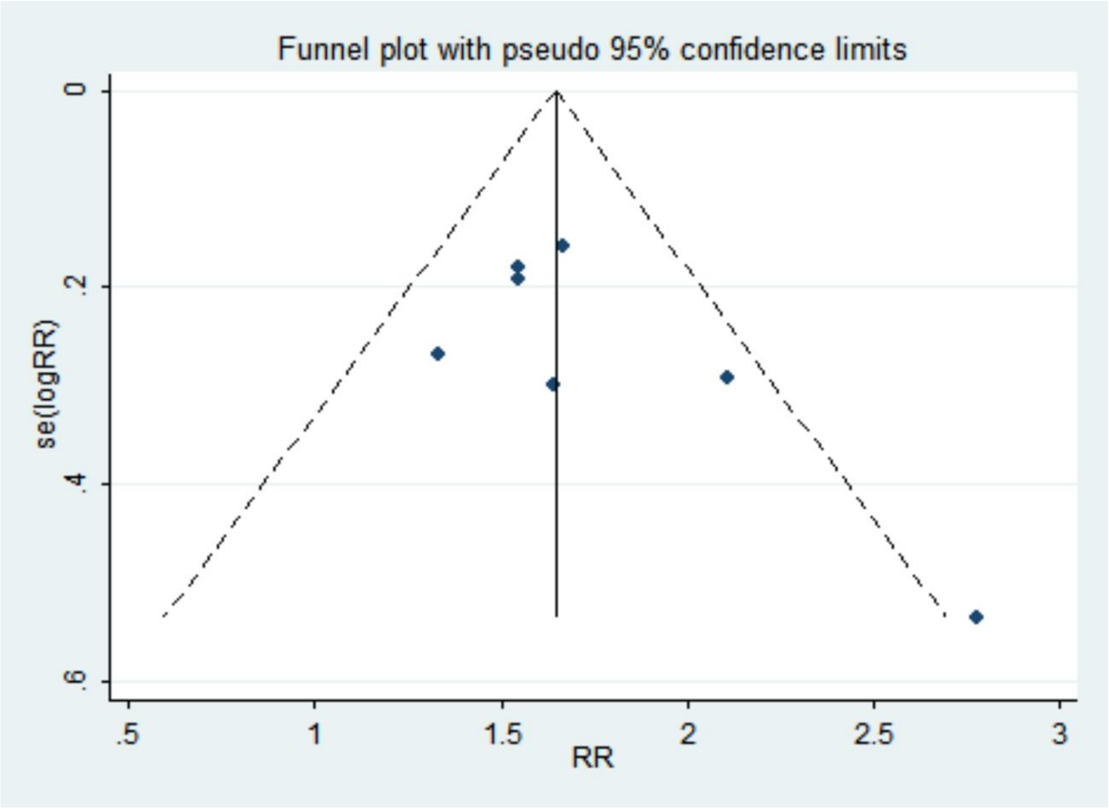


**Supplement Figure 23** Begg's for SAEs

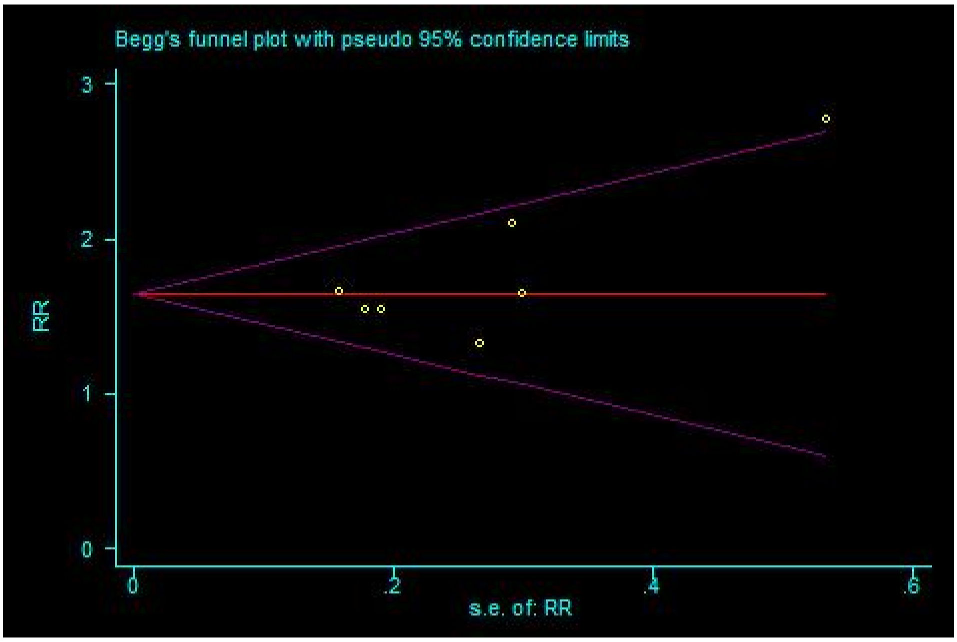

**Supplement Figure 24** Egger's for SAEs

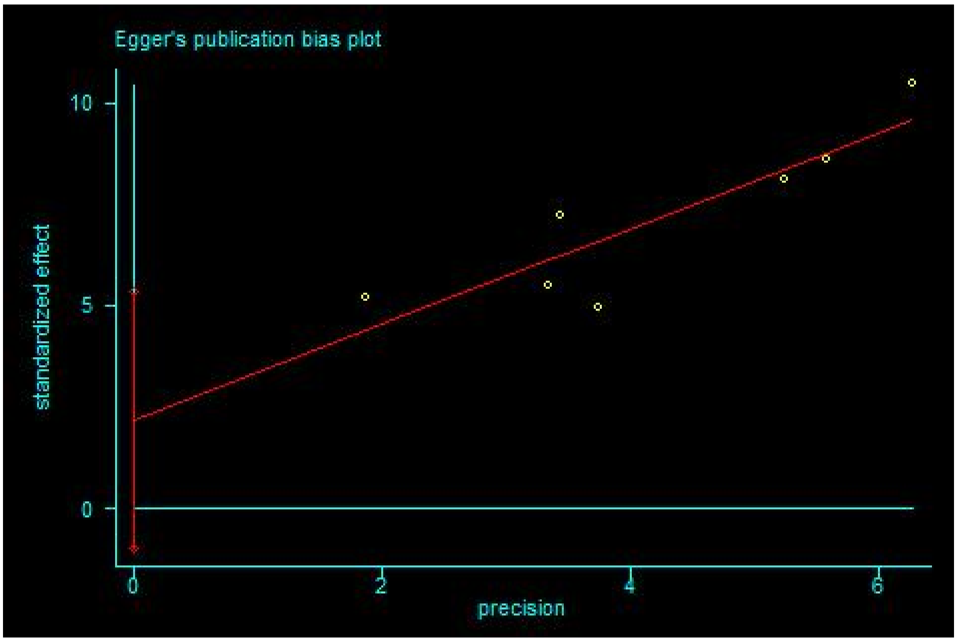


**Supplement Figure 25** Tests of publication bias for SAEs

```
Tests for Publication Bias

Begg's Test

  adj. Kendall's Score (P-Q) =       7
          Std. Dev. of Score =    6.66
           Number of Studies =       7
                          z  =    1.05
                    Pr > |z| =   0.293
                          z  =    0.90 (continuity corrected)
                    Pr > |z| =   0.368 (continuity corrected)

Egger's test
```

| Std_Eff | Coef. | Std. Err. | t | P>\|t\| | [95% Conf. | Interval] |
|---|---|---|---|---|---|---|
| slope | 1.178202 | .2780938 | 4.24 | 0.008 | .4633395 | 1.893065 |
| bias | 2.191876 | 1.236762 | 1.77 | 0.137 | -.987321 | 5.371073 |

**Supplement Figure 26 Forest plot of the meta-analysis for ORR (CDK4/6 i drug classification subgroup analysis)**

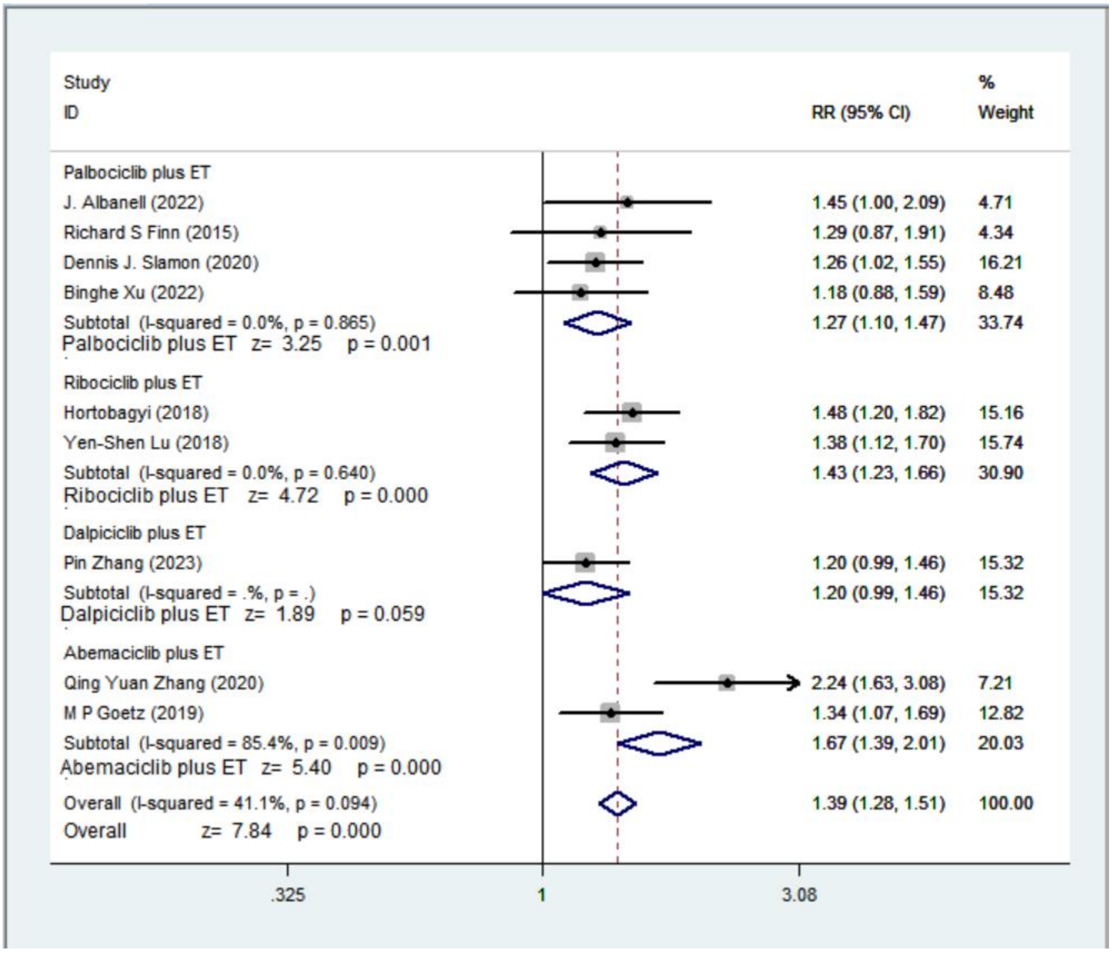

**Supplement Figure 27 Forest plot of the meta-analysis for DCR (CDK4/6 i drug classification subgroup analysis)**

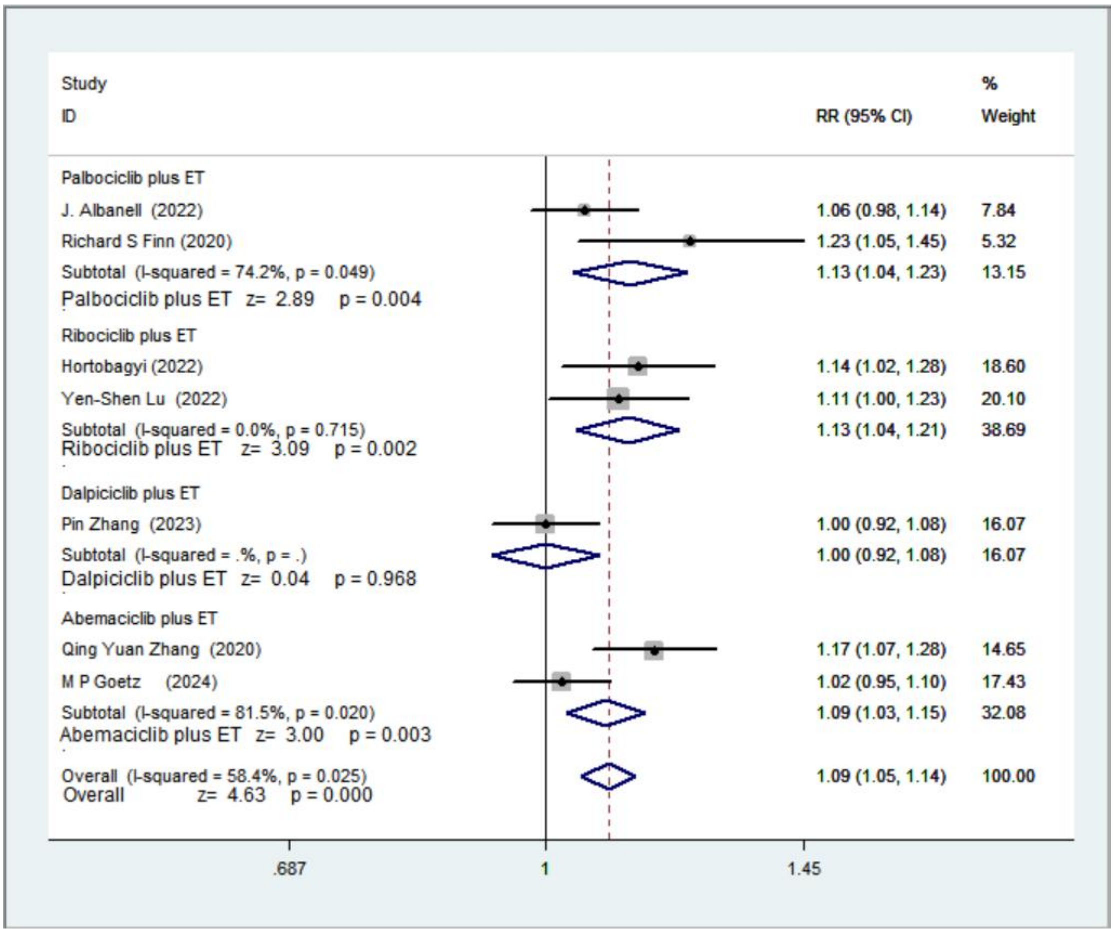

**Supplement Figure 28 Forest plot of the meta-analysis for PFS (CDK4/6 i drug classification subgroup analysis)**

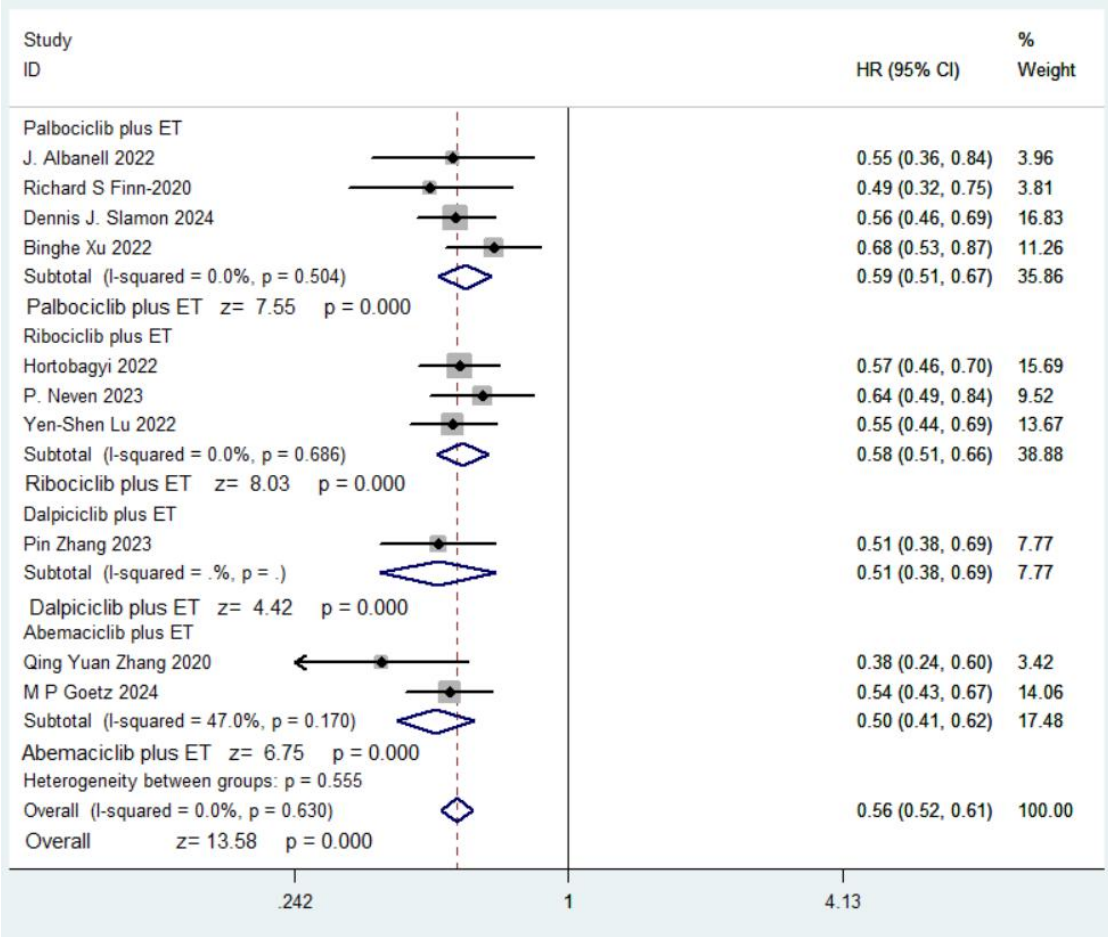

**Supplement Figure 29 Forest plot of the meta-analysis for OS (CDK4/6 i drug classification subgroup analysis)**

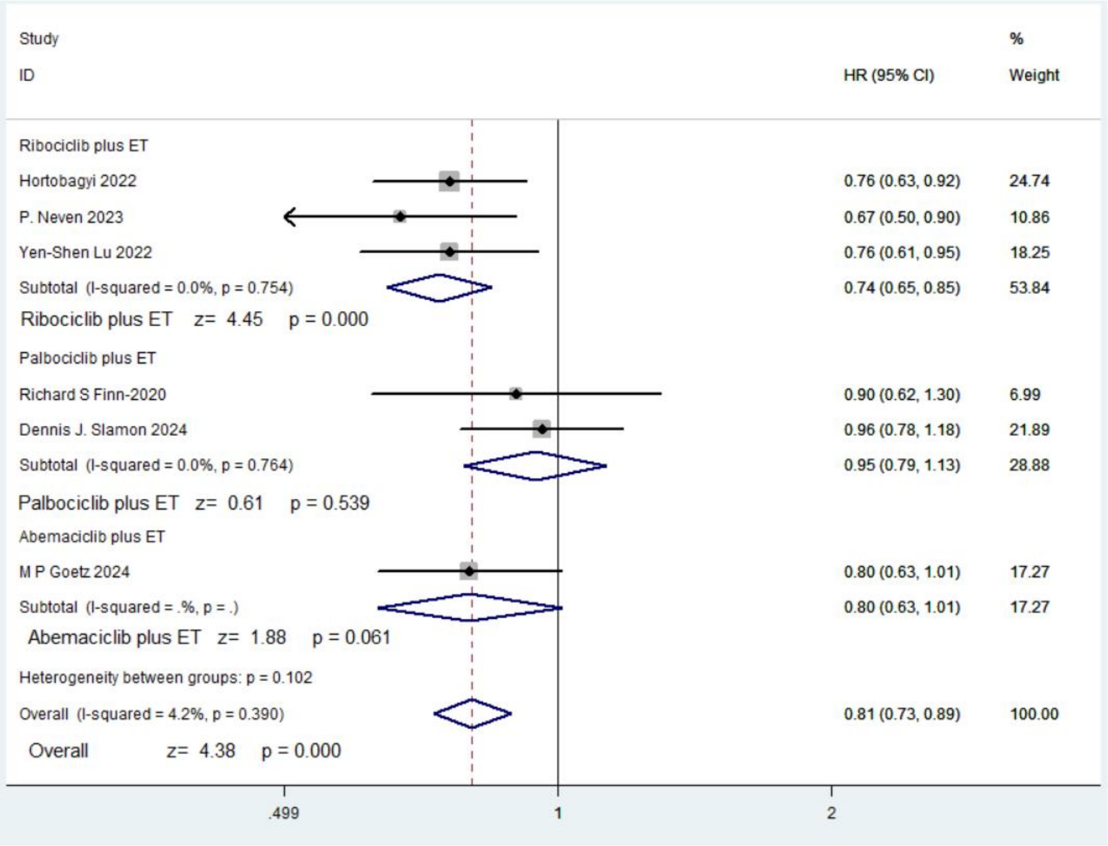

**Supplement Figure 30 Forest plot of the meta-analysis for ORR (subgroup analysis of menopausal status)**

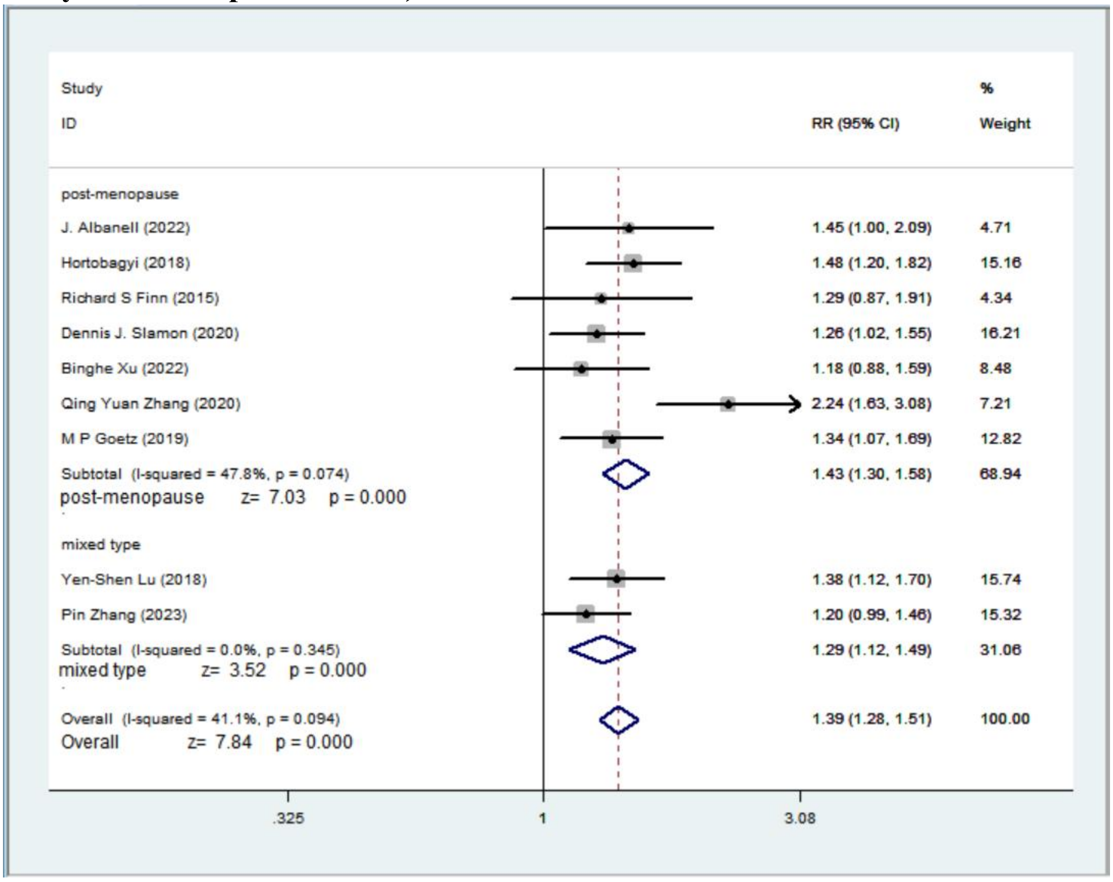

**Supplement Figure 31 Forest plot of the meta-analysis for DCR (subgroup analysis of menopausal status)**

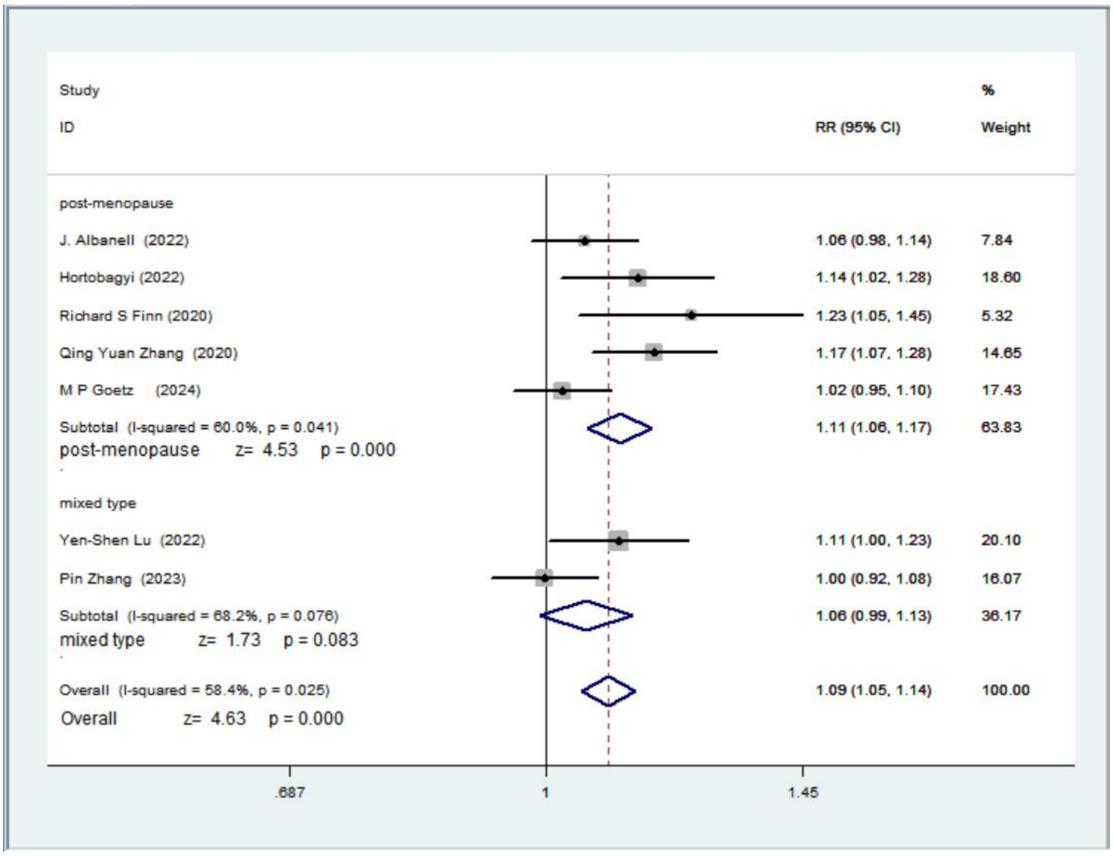

**Supplement Figure 32 Forest plot of the meta-analysis for PFS (subgroup analysis of menopausal status)**

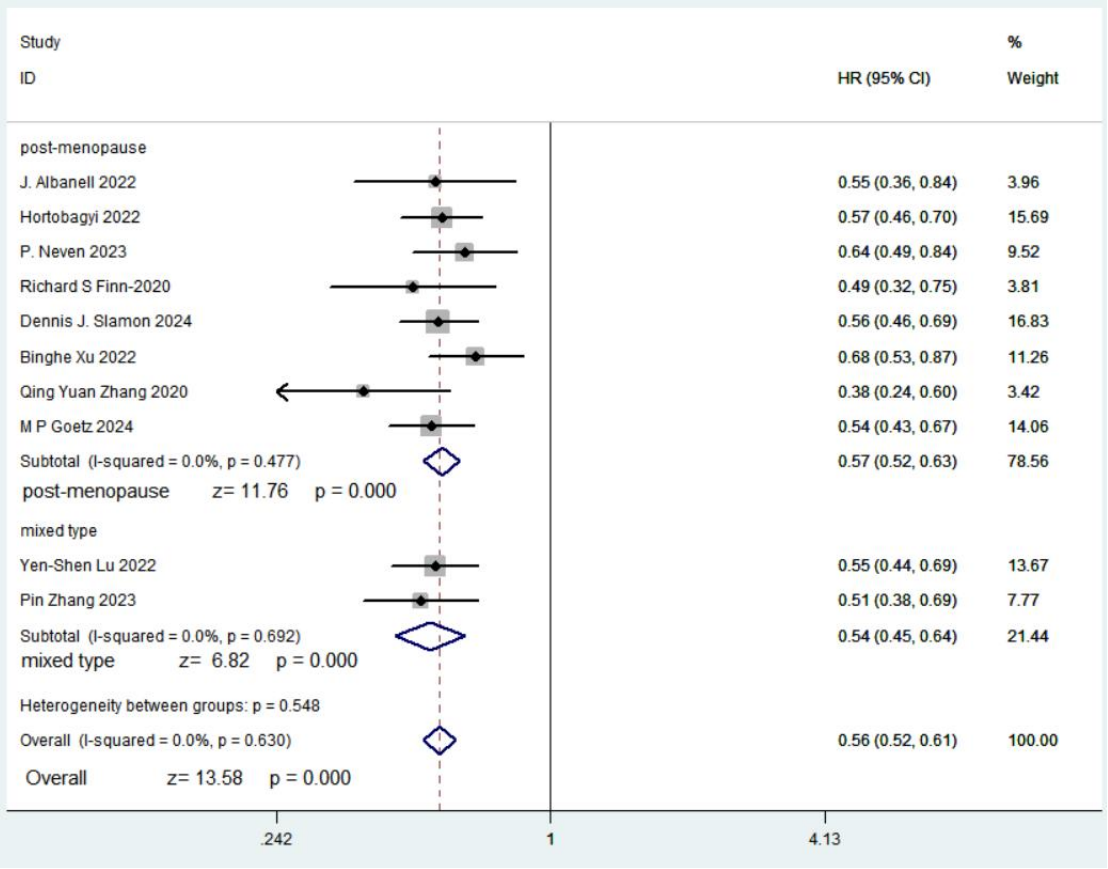

**Supplement Figure 33 Forest plot of the meta-analysis for OS (subgroup analysis of menopausal status)**

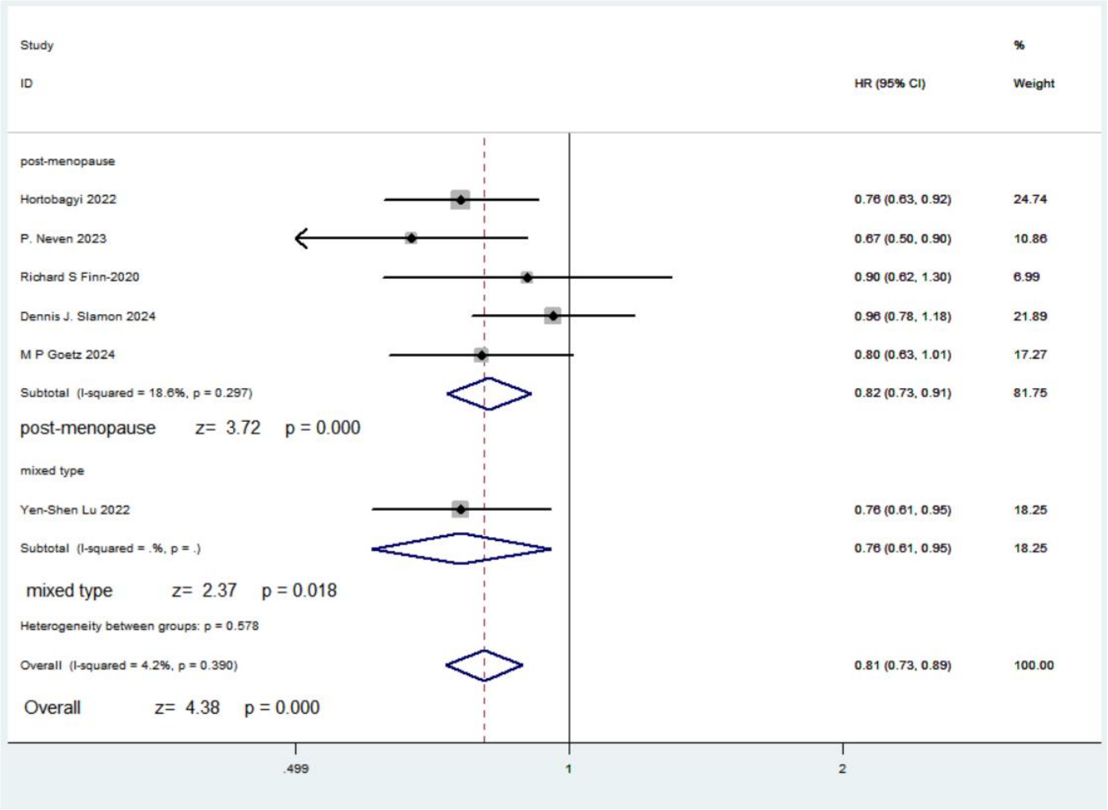

Supplement: Supplementary file 1 [file DataSheet2.pdf]
