# Supplementary material for: Comparative long-term outcomes of first-line CDK4/6 inhibitors plus endocrine therapy versus endocrine therapy in patients with HR+/HER2-metastatic or advanced breast cancer: a meta-analysis
Source: Front Pharmacol. 2025 Jul 25;16:1600892. doi: 10.3389/fphar.2025.1600892 (PMC12331727; doi:10.3389/fphar.2025.1600892)
Supplement: Supplementary file 2 [file DataSheet1.pdf]

Comprehensive listing of the search results.

PubMed		
No.	Query	Results
#1	(((((((((((((((((((((((((((((((((((((Breast Neoplasms[Title/Abstract]) OR (Breast Neoplasm[Title/Abstract])) OR (Neoplasm, Breast[Title/Abstract])) OR (Breast Tumors[Title/Abstract])) OR (Breast Tumor[Title/Abstract])) OR (Tumor, Breast[Title/Abstract])) OR (Tumors, Breast[Title/Abstract])) OR (Neoplasms, Breast[Title/Abstract])) OR (Breast Cancer[Title/Abstract])) OR (Cancer, Breast[Title/Abstract])) OR (Mammary Cancer[Title/Abstract])) OR (Cancer, Mammary[Title/Abstract])) OR (Cancers, Mammary[Title/Abstract])) OR (Mammary Cancers[Title/Abstract])) OR (Malignant Neoplasm of Breast[Title/Abstract])) OR (Breast Malignant Neoplasm[Title/Abstract])) OR (Breast Malignant Neoplasms[Title/Abstract])) OR (Malignant Tumor of Breast[Title/Abstract])) OR (Breast Malignant Tumor[Title/Abstract])) OR (Breast Malignant Tumors[Title/Abstract])) OR (Cancer of Breast[Title/Abstract])) OR (Cancer of the Breast[Title/Abstract])) OR (Mammary Carcinoma, Human[Title/Abstract])) OR (Carcinoma, Human Mammary[Title/Abstract])) OR (Carcinomas, Human Mammary[Title/Abstract])) OR (Human Mammary Carcinomas[Title/Abstract])) OR (Mammary Carcinomas, Human[Title/Abstract])) OR (Human Mammary Carcinoma[Title/Abstract])) OR (Mammary Neoplasms, Human[Title/Abstract])) OR (Human Mammary Neoplasm[Title/Abstract])) OR (Human Mammary Neoplasms[Title/Abstract])) OR (Neoplasm, Human Mammary[Title/Abstract])) OR (Neoplasms, Human Mammary[Title/Abstract])) OR (Mammary Neoplasm, Human[Title/Abstract])) OR (Breast Carcinoma[Title/Abstract])) OR (Breast Carcinomas[Title/Abstract])) OR (Carcinoma, Breast[Title/Abstract])) OR (Carcinomas, Breast[Title/Abstract])	383182
#2	（metastatic[Title/Abstract]） OR （advanced[Title/Abstract]）	818651
#3	(((((First-line treatment[Title/Abstract]) OR (First-line therapy[Title/Abstract])) OR (First-line[Title/Abstract])) OR (First line[Title/Abstract])) OR (Untreated[Title/Abstract])) OR (Naive[Title/Abstract])	413864
#4	((((((((((((CDK4/6[Title/Abstract]) OR (CDK4/6 inhibitors[Title/Abstract])) OR (CDK4/6 inhibitor[Title/Abstract])) OR (Cyclin-dependent kinase 4/6 inhibitors[Title/Abstract])) OR (Cyclin-dependent kinase 4/6 inhibitor[Title/Abstract])) OR (Abemaciclib[Title/Abstract])) OR (abemaciclib mesylate[Title/Abstract])) OR (Verzenio[Title/Abstract])) OR (Palbociclib[Title/Abstract])) OR (Ibrance[Title/Abstract])) OR (Ribociclib[Title/Abstract])) OR (LEE011[Title/Abstract])) OR (Kisqali[Title/Abstract])	4302
#5	(((endocrine[Title/Abstract]) OR (endocrine therapy[Title/Abstract])) OR (endocrine treatment[Title/Abstract])) OR (Selective Estrogen Receptor Modulators[Title/Abstract])) OR (SERMs[Title/Abstract])) OR (Estrogen Receptor Modulators, Selective[Title/Abstract])) OR (Selective Estrogen Receptor Modulator[Title/Abstract])) OR (SERM[Title/Abstract])) OR (Estrogen Receptor Modulator, Selective[Title/Abstract])) OR (Tamoxifen[Title/Abstract])) OR (Nolvadex[Title/Abstract]))	196997

	OR (Novaldex[Title/Abstract])) OR (Tamoxifen Citrate[Title/Abstract])) OR (Citrate, Tamoxifen[Title/Abstract])) OR (Zitazonium[Title/Abstract])) OR (Toremifene[Title/Abstract])) OR (Toremifene Citrate[Title/Abstract])) OR (Citrate, Toremifene[Title/Abstract])) OR (Raloxifene[Title/Abstract])) OR (Raloxifene Hydrochloride[Title/Abstract])) OR (Raloxifene HCl[Title/Abstract])) OR (ovarian function suppression[Title/Abstract])) OR (Goserelin[Title/Abstract])) OR (Zoladex[Title/Abstract])) OR (Goserelin Acetate[Title/Abstract])) OR (Acetate, Goserelin[Title/Abstract])) OR (Triptorelin[Title/Abstract])) OR (Triptorelin Pamoate[Title/Abstract])) OR (Pamoate, Triptorelin[Title/Abstract])) OR (Leuprolide[Title/Abstract])) OR (Leuprorelin[Title/Abstract])) OR (Leuprolide Acetate[Title/Abstract])) OR (Acetate, Leuprolide[Title/Abstract])) OR (Leuprolide Monoacetate[Title/Abstract])) OR (Monoacetate, Leuprolide[Title/Abstract])) OR (Lupron[Title/Abstract])) OR (aromatase inhibitor[Title/Abstract])) OR (Inhibitors, Aromatase[Title/Abstract])) OR (Aromatase Inhibitor[Title/Abstract])) OR (Inhibitor, Aromatase[Title/Abstract])) OR (Aminoglutethimide[Title/Abstract])) OR (Anastrozole[Title/Abstract])) OR (Anastrazole[Title/Abstract])) OR (Arimidex[Title/Abstract])) OR (Letrozole[Title/Abstract])) OR (Exemestane[Title/Abstract])) OR (examestane[Title/Abstract])) OR (selective estrogen receptor degrader[Title/Abstract])) OR (SERD[Title/Abstract])) OR (SERDS[Title/Abstract])) OR (fulvestrant[Title/Abstract])) OR (Faslodex[Title/Abstract])) OR (Megestrol[Title/Abstract])	
#6	((((((randomized controlled trial[Title/Abstract]) OR (controlled clinical trial[Title/Abstract])) OR (Randomized[Title/Abstract])) OR (placebo[Title/Abstract])) OR (clinical trials as topic[Title/Abstract])) OR (randomly[Title/Abstract])) OR (Trial[Title/Abstract])	1543676
#7	#1 AND #2 AND #3 AND #4 AND #5 AND #6	170

Web of science		
No.	Query	Results
#1	(((((((((((((((((((((((((((((((((((((TS=(Breast Neoplasms)) OR TS=(Breast Neoplasm)) OR TS=(Neoplasm, Breast)) OR TS=(Breast Tumors)) OR TS=(Breast Tumor)) OR TS=(Tumor, Breast)) OR TS=(Tumors, Breast)) OR TS=(Neoplasms, Breast)) OR TS=(Breast Cancer)) OR TS=(Cancer, Breast)) OR TS=(Mammary Cancer)) OR TS=(Cancer, Mammary)) OR TS=(Cancers, Mammary)) OR TS=(Mammary Cancers)) OR TS=(Malignant Neoplasm of Breast)) OR TS=(Breast Malignant Neoplasm)) OR TS=(Breast Malignant Neoplasms)) OR TS=(Malignant Tumor of Breast)) OR TS=(Breast Malignant Tumor)) OR TS=(Breast Malignant Tumors)) OR TS=(Cancer of Breast)) OR TS=(Cancer of the Breast)) OR TS=(Mammary Carcinoma, Human)) OR TS=(Carcinoma, Human Mammary)) OR TS=(Carcinomas, Human Mammary)) OR TS=(Human Mammary Carcinomas)) OR TS=(Mammary Carcinomas, Human)) OR TS=(Human Mammary Carcinoma)) OR TS=(Mammary Neoplasms, Human)) OR TS=(Human Mammary Neoplasm)) OR TS=(Human Mammary Neoplasms)) OR TS=(Neoplasm, Human Mammary)) OR TS=(Neoplasms, Human Mammary)) OR TS=(Mammary Neoplasm, Human)) OR TS=(Breast Carcinoma)) OR TS=(Breast Carcinomas)) OR TS=(Carcinoma, Breast)) OR TS=(Carcinomas, Breast)	1224405
#2	(TS=(advanced)) OR TS=(metastatic)	3436191
#3	(((((TS=(First-line treatment)) OR TS=(First-line therapy)) OR TS=(First-line)) OR TS=(First line)) OR TS=(Untreated)) OR TS=(Naive)	2316339
#4	(((((((((((TS=(CDK4/6)) OR TS=(CDK4/6 inhibitors)) OR TS=(CDK4/6 inhibitor)) OR TS=(Cyclin-dependent kinase 4/6 inhibitors)) OR TS=(Cyclin-dependent kinase 4/6 inhibitor)) OR TS=(Abemaciclib)) OR TS=(abemaciclib mesylate)) OR TS=(Verzenio)) OR TS=(Palbociclib)) OR TS=(Palbociclib)) OR TS=(Ribociclib)) OR TS=(LEE011)) OR TS=(Kisqali)	10970
#5	(((TS=(endocrine)) OR TS=(endocrine therapy)) OR TS=(endocrine treatment)) OR TS=(Selective Estrogen Receptor Modulators)) OR TS=(SERMs)) OR TS=(Estrogen Receptor Modulators, Selective)) OR TS=(Selective Estrogen Receptor Modulator)) OR TS=(SERM)) OR TS=(Estrogen Receptor Modulator, Selective)) OR TS=(Tamoxifen)) OR TS=(Nolvadex)) OR TS=(Novaldex)) OR TS=(Tamoxifen Citrate)) OR TS=(Citrate, Tamoxifen)) OR TS=(Zitazonium)) OR TS=(Toremifene)) OR TS=(Toremifene Citrate)) OR TS=(Citrate, Toremifene)) OR TS=(Raloxifene)) OR TS=(Raloxifene Hydrochloride)) OR TS=(Raloxifene HCl)) OR TS=(ovarian function suppression)) OR TS=(Goserelin)) OR TS=(Zoladex)) OR TS=(Goserelin Acetate)) OR TS=(Acetate, Goserelin)) OR TS=(Triptorelin)) OR TS=(Triptorelin Pamoate)) OR TS=(Pamoate, Triptorelin)) OR TS=(Leuprolide)) OR TS=(Leuprorelin)) OR TS=(Leuprolide Acetate)) OR TS=(Acetate, Leuprolide)) OR	3987020

	TS=(Leuprolide Monoacetate)) OR TS=(Monoacetate, Leuprolide)) OR TS=(Lupron)) OR TS=(aromatase inhibitor)) OR TS=(Inhibitors, Aromatase)) OR TS=(Aromatase Inhibitor)) OR TS=(Inhibitor, Aromatase)) OR TS=(Aminoglutethimide)) OR TS=(Anastrozole)) OR TS=(Anastrazole)) OR TS=(Arimidex)) OR TS=(Letrozole)) OR TS=(Exemestane)) OR TS=(examestane)) OR TS=(selective estrogen receptor degrader)) OR TS=(SERD)) OR TS=(SERDS)) OR TS=(fulvestrant)) OR TS=(Faslodex)) OR TS=(Megestrol)	
#6	((((((TS=(randomized controlled trial)) OR TS=(controlled clinical trial)) OR TS=(Randomized)) OR TS=(placebo)) OR TS=(clinical trials as topic)) OR TS=(randomly)) OR TS=(Trial)	4126845
#7	#1 AND #2 AND #3 AND #4 AND #5 AND #6	443

Cochrane library		
No.	Query	Results
#1 乳腺癌	(Breast Neoplasms):ti,ab,kw OR (Breast Neoplasm):ti,ab,kw OR (Neoplasm, Breast):ti,ab,kw OR (Breast Tumors):ti,ab,kw OR (Breast Tumor):ti,ab,kw OR (Tumor, Breast):ti,ab,kw OR (Tumors, Breast):ti,ab,kw OR (Neoplasms, Breast):ti,ab,kw OR (Breast Cancer):ti,ab,kw OR (Cancer, Breast):ti,ab,kw OR (Mammary Cancer):ti,ab,kw OR (Cancer, Mammary):ti,ab,kw OR (Cancers, Mammary):ti,ab,kw OR (Mammary Cancers):ti,ab,kw OR (Malignant Neoplasm of Breast):ti,ab,kw OR (Breast Malignant Neoplasm):ti,ab,kw OR (Breast Malignant Neoplasms):ti,ab,kw OR (Malignant Tumor of Breast):ti,ab,kw OR (Breast Malignant Tumor):ti,ab,kw OR (Breast Malignant Tumors):ti,ab,kw OR (Cancer of Breast):ti,ab,kw OR (Cancer of the Breast):ti,ab,kw OR (Mammary Carcinoma, Human):ti,ab,kw OR (Carcinoma, Human Mammary):ti,ab,kw OR (Carcinomas, Human Mammary):ti,ab,kw OR (Human Mammary Carcinomas):ti,ab,kw OR (Mammary Carcinomas, Human):ti,ab,kw OR (Human Mammary Carcinoma):ti,ab,kw OR (Mammary Neoplasms, Human):ti,ab,kw OR (Human Mammary Neoplasm):ti,ab,kw OR (Human Mammary Neoplasms):ti,ab,kw OR (Neoplasm, Human Mammary):ti,ab,kw OR (Neoplasms, Human Mammary):ti,ab,kw OR (Mammary Neoplasm, Human):ti,ab,kw OR (Breast Carcinoma):ti,ab,kw OR(Breast Carcinomas):ti,ab,kw OR (Carcinoma, Breast):ti,ab,kw OR (Carcinomas, Breast):ti,ab,kw	46763 Results
#2	(metastatic):ti,ab,kw OR (advanced):ti,ab,kw	90298 Results
#3	(First-line treatment):ti,ab,kw OR (First-line therapy):ti,ab,kw OR (First-line):ti,ab,kw OR (First line):ti,ab,kw OR (Untreated):ti,ab,kw OR (Naive):ti,ab,kw	72903 Results
#4	(CDK4/6):ti,ab,kw OR (CDK4/6 inhibitors):ti,ab,kw OR (CDK4/6 inhibitor):ti,ab,kw OR (Cyclin-dependent kinase 4/6 inhibitors):ti,ab,kw OR (Cyclin-dependent kinase 4/6 inhibitor):ti,ab,kw OR (Abemaciclib):ti,ab,kw OR (abemaciclib mesylate):ti,ab,kw OR (Verzenio):ti,ab,kw OR (Palbociclib):ti,ab,kw OR (Ibrance):ti,ab,kw OR (Ribociclib):ti,ab,kw OR (LEE011):ti,ab,kw OR (Kisqali):ti,ab,kw	1407 Results

#5	(Endocrine):ti,ab,kw OR (endocrine therapy):ti,ab,kw OR (endocrine treatment):ti,ab,kw OR (Selective Estrogen Receptor Modulators):ti,ab,kw OR (SERMs):ti,ab,kw OR (Estrogen Receptor Modulators, Selective):ti,ab,kw OR (Selective Estrogen Receptor Modulator):ti,ab,kw OR (SERM):ti,ab,kw OR (Estrogen Receptor Modulator, Selective):ti,ab,kw OR (Tamoxifen):ti,ab,kw OR (Nolvadex):ti,ab,kw OR (Novaldex):ti,ab,kw OR (Tamoxifen Citrate):ti,ab,kw OR (Citrate, Tamoxifen):ti,ab,kw OR (Zitazonium):ti,ab,kw OR (Toremifene):ti,ab,kw OR (Toremifene Citrate):ti,ab,kw OR (Citrate, Toremifene):ti,ab,kw OR (Raloxifene):ti,ab,kw OR (Raloxifene Hydrochloride):ti,ab,kw OR (Raloxifene HCl):ti,ab,kw OR (ovarian function suppression):ti,ab,kw OR (Goserelin):ti,ab,kw OR (Zoladex):ti,ab,kw OR (Goserelin Acetate):ti,ab,kw OR (Acetate, Goserelin):ti,ab,kw OR (Triptorelin):ti,ab,kw OR (Triptorelin Pamoate):ti,ab,kw OR (Pamoate, Triptorelin):ti,ab,kw OR (Leuprolide):ti,ab,kw OR (Leuprorelin):ti,ab,kw OR (Leuprolide Acetate):ti,ab,kw OR (Acetate, Leuprolide):ti,ab,kw OR (Leuprolide Monoacetate):ti,ab,kw OR (Monoacetate, Leuprolide):ti,ab,kw OR (Lupron):ti,ab,kw OR (aromatase inhibitor):ti,ab,kw OR (Inhibitors, Aromatase):ti,ab,kw OR (Aromatase Inhibitor):ti,ab,kw OR (Inhibitor, Aromatase):ti,ab,kw OR (Aminoglutethimide):ti,ab,kw OR (Anastrozole):ti,ab,kw OR (Anastrazole):ti,ab,kw OR (Arimidex):ti,ab,kw OR (Letrozole):ti,ab,kw OR (Exemestane):ti,ab,kw OR (examestane):ti,ab,kw OR (selective estrogen receptor degrader):ti,ab,kw OR (SERD):ti,ab,kw OR (SERDS):ti,ab,kw OR (fulvestrant):ti,ab,kw OR (Faslodex):ti,ab,kw OR (Megestrol):ti,ab,kw	25367 Results
#6	(randomized controlled trial):ti,ab,kw OR (controlled clinical trial):ti,ab,kw OR (Randomized):ti,ab,kw OR (placebo):ti,ab,kw OR (clinical trials as topic):ti,ab,kw OR (randomly):ti,ab,kw OR (Trial):ti,ab,kw	1480902 Results
#7	#1 AND #2 AND #3 AND #4 AND #5 AND #6	378 Results

Embase

No.	Query	Results
#1	('Breast Neoplasms'):ab,ti OR (('Breast Neoplasm'):ab,ti) OR (('Neoplasm, Breast'):ab,ti) OR (('Breast Tumors'):ab,ti) OR (('Breast Tumor'):ab,ti) OR (('Tumor, Breast'):ab,ti) OR (('Tumors, Breast'):ab,ti) OR (('Neoplasms, Breast'):ab,ti) OR (('Breast Cancer'):ab,ti) OR (('Cancer, Breast'):ab,ti) OR (('Mammary Cancer'):ab,ti) OR (('Cancer, Mammary'):ab,ti) OR (('Cancers, Mammary'):ab,ti) OR (('Mammary Cancers'):ab,ti) OR (('Malignant Neoplasm of Breast'):ab,ti) OR (('Breast Malignant Neoplasm'):ab,ti) OR (('Breast Malignant Neoplasms'):ab,ti) OR (('Malignant Tumor of Breast'):ab,ti) OR (('Breast Malignant Tumor'):ab,ti) OR (('Breast Malignant Tumors'):ab,ti) OR (('Cancer of Breast'):ab,ti) OR (('Cancer of the Breast'):ab,ti) OR (('Mammary Carcinoma, Human'):ab,ti) OR (('Carcinoma, Human Mammary'):ab,ti) OR (('Human Mammary Carcinomas'):ab,ti) OR (('Human Mammary Carcinoma'):ab,ti) OR (('Human Mammary Neoplasms'):ab,ti) OR (('Breast Carcinoma'):ab,ti) OR (('Breast Carcinomas'):ab,ti) OR (('Carcinoma, Breast'):ab,ti) OR (('Carcinomas, Breast'):ab,ti) OR ('Carcinomas, Human Mammary'):ab,ti OR (('Mammary Carcinomas, Human'):ab,ti) OR (('Mammary Neoplasms, Human'):ab,ti) OR (('Human Mammary Neoplasm'):ab,ti) OR (('Neoplasm, Human Mammary'):ab,ti) OR (('Neoplasms, Human Mammary'):ab,ti) OR (('Mammary Neoplasm, Human'):ab,ti) OR ('Carcinomas, Human Mammary'):ab,ti OR (('Mammary Carcinomas, Human'):ab,ti) OR (('Mammary Neoplasms, Human'):ab,ti) OR (('Human Mammary Neoplasm'):ab,ti) OR (('Neoplasm, Human Mammary'):ab,ti) OR (('Neoplasms, Human Mammary'):ab,ti) OR (('Mammary Neoplasm, Human'):ab,ti)	541023
#2	(metastatic):ab,ti OR ((advanced):ab,ti)	1225563
#3	('First-line treatment'):ab,ti OR (('First-line therapy'):ab,ti) OR ((First-line):ab,ti) OR (('First line'):ab,ti) OR ((Untreated):ab,ti) OR ((Naive):ab,ti)	640074
# 4	('CDK4/6'):ab,ti OR (('CDK4/6 inhibitors'):ab,ti) OR (('CDK4/6 inhibitor'):ab,ti) OR (('Cyclin-dependent kinase 4/6 inhibitors'):ab,ti) OR (('Cyclin-dependent kinase 4/6 inhibitor'):ab,ti) OR ((Abemaciclib):ab,ti) OR (('abemaciclib mesylate'):ab,ti) OR ((Verzenio):ab,ti) OR ((Palbociclib):ab,ti) OR ((Ibrance):ab,ti) OR ((ribociclib):ab,ti) OR ((LEE011):ab,ti) OR ((Kisqali):ab,ti)	8950
#5	(endocrine):ab,ti OR (('endocrine therapy'):ab,ti) OR (('endocrine treatment'):ab,ti) OR (('Selective Estrogen Receptor Modulators'):ab,ti) OR ((SERMs):ab,ti) OR (('Estrogen Receptor Modulators, Selective'):ab,ti) OR (('Selective Estrogen Receptor Modulator'):ab,ti) OR ((SERM):ab,ti) OR (('Estrogen Receptor Modulator,	273276

	Selective'):ab,ti) OR ((Tamoxifen):ab,ti) OR ((Nolvadex):ab,ti) OR ((Novaldex):ab,ti) OR (('Tamoxifen Citrate'):ab,ti) OR (('Citrate, Tamoxifen'):ab,ti) OR ((Zitazonium):ab,ti) OR ((Toremifene):ab,ti) OR (('Toremifene Citrate'):ab,ti) OR (('Citrate, Toremifene'):ab,ti) OR ((Raloxifene):ab,ti) OR (('Raloxifene Hydrochloride'):ab,ti) OR (('Raloxifene HCl'):ab,ti) OR (('ovarian function suppression'):ab,ti) OR ((Goserelin):ab,ti) OR ((Zoladex):ab,ti) OR (('Goserelin Acetate'):ab,ti) OR (('Acetate, Goserelin'):ab,ti) OR ((Triptorelin):ab,ti) OR (('Triptorelin Pamoate'):ab,ti) OR (('Pamoate, Triptorelin'):ab,ti) OR ((Leuprolide):ab,ti) OR ((Leuprorelin):ab,ti) OR (('Leuprolide Acetate'):ab,ti) OR (('Acetate, Leuprolide'):ab,ti) OR (('Leuprolide Monoacetate'):ab,ti) OR (('Monoacetate, Leuprolide'):ab,ti) OR ((Lupron):ab,ti) OR (('aromatase inhibitor'):ab,ti) OR (('Inhibitors, Aromatase'):ab,ti) OR (('Aromatase Inhibitor'):ab,ti) OR (('Inhibitor, Aromatase'):ab,ti) OR ((Aminoglutethimide):ab,ti) OR ((Anastrozole):ab,ti) OR ((Anastrazole):ab,ti) OR ((Arimidex):ab,ti) OR ((Letrozole):ab,ti) OR ((Exemestane):ab,ti) OR ((examestane):ab,ti) OR (('selective estrogen receptor degrader'):ab,ti) OR ((SERD):ab,ti) OR ((SERDS):ab,ti) OR ((fulvestrant):ab,ti) OR ((Faslodex):ab,ti) OR ((Megestrol):ab,ti)	
#6	('randomized controlled trial'):ab,ti OR (('controlled clinical trial'):ab,ti) OR ((Randomized):ab,ti) OR ((placebo):ab,ti) OR (('clinical trials as topic'):ab,ti) OR ((randomly):ab,ti) OR ((Trial):ab,ti)	2199499
#7	#1 AND #2 AND #3 AND #4 AND #5 AND #6	602
